# Novel forms for the expression of aspect in heritage Greek across majority languages

**DOI:** 10.1371/journal.pone.0319154

**Published:** 2025-05-15

**Authors:** Vasiliki Rizou, Despina Papadopoulou, Onur Özsoy, Artemis Alexiadou

**Affiliations:** 1 Department of German Studies and Linguistics, Institute of German Language and Linguistics, Humboldt University, Berlin, Germany; 2 School of Philology, Linguistics, Aristotle University, Thessaloniki, Greece; 3 Leibniz-Center General Linguistics, Berlin, Germany; University of Missouri Columbia, UNITED STATES OF AMERICA

## Abstract

This paper investigates, on the one hand, which verbal features are re-organized in heritage grammars and, on the other hand, the production of novel non-canonical forms for the expression of verbal aspect by Greek heritage speakers in Germany and the US compared to monolingually-raised speakers of Modern Greek (henceforth Greek). As aspect cannot be seen in isolation from other morphological features such as voice, tense, and phi-features, the analysis of the novel forms is conducted under the prism of the verbal complex. The results indicate that φ-features and aspect seem to be the most re-organized elements encoded in Greek verbs and, furthermore, that heritage speaker groups differ significantly from monolingually-raised controls in terms of the production of novel morphological forms, demonstrating that heritage speakers, especially those in the US, face difficulties with the morpho-phonological adjustments needed to be built in verbal forms.

## Introduction

Aspect has been extensively examined in diverse languages and represents one of the primary areas that are studied in research related to heritage languages [1–3]. Aspect is regarded as one of the most assailable phenomena affected by language contact, particularly in languages with concatenate morphology, such as Greek. The aim of the present study is twofold. First, it investigates the verbal features that might undergo re-organization while constructing a verbal form in Greek. Second, morphologically novel verbs in heritage speakers’ grammar are explored through an elicited production task. Although several experimental studies have explored heritage speakers’ accuracy on the use of aspect, this study analyzes the composita of the verbal forms that are susceptible to change. More specifically, heritage speakers’ verbal productions are explored through an error analysis and the morphologically novel forms are categorized according to the features that appear to be precarious to change.

The study is conducted on a relatively new emerging population which can be found in the literature as heritage speakers. Heritage speakers are minority language speakers who grew up in a majority language environment. These speakers are bilinguals acquiring either simultaneously or sequentially both their heritage language and the language of their larger society in a natural setting and thus, they are considered to be in the spectrum of a native speaker [4–5]. Furthermore, another characteristic of these speakers is that their proficiency in the heritage language declines over time, meaning that during their puberty and adulthood heritage speakers tend to be dominant in the language of their larger national community [6]. The age of onset to bilingualism, parental input, proficiency and language dominance in both languages, literacy and formal education are some factors compiling the speakers’ linguistic profile that affect their performance on different phenomena in the heritage language [7–14 among others]. The emergence of such bilingual groups results usually from economic and political immigration. The study focuses on two different groups of heritage speakers, namely Greek heritage speakers in the US recruited in New York, NY and Chicago, IL and heritage speakers in Germany recruited in Berlin.

According to Comrie [15], aspect reveals the internal temporal constituency of a situation (situation-internal time) and distinguishes between grammatical (view-point) and lexical (situation/Aktionsart). Grammatical aspect which is morphologically encoded on the verb distinguishes between perfective and imperfective. The former indicates the view of a situation as a whole while the latter pays attention to the internal structure of the situation [15]. The lexical aspect refers to the general inherent semantic oppositions of the verbs and is the lexicalization of the semantic distinctions of the different verb classes (states, activities, accomplishments, achievements) as these are defined by Vendler [16] and, elaborated by Smith [17]. The primary and well-attested theory on the acquisition of aspect in L1 and L2, the Aspect Hypothesis, predicts that the lexical aspect strongly influences learners in acquiring tense and aspect markers (see [18] for Russian children). Furthermore, past perfective markers are associated with telic verbs (achievements and accomplishments), while general imperfective/ progressive markers are associated with atelic verbs (states and activities) [19–20], although it has been disputed for some languages based on the input-based explanation (cf. for Mandarin Chinese: [20–21]). L1 Greek children acquire early the morphological marking of perfective aspect -already at 1;1 years old, according to Stephany [22] - despite the morpho-phonological rules applied to the verb stem and thus, the perfective aspect is considered the default in Greek [23]. In contrast,the imperfective aspect, which in Greek denotes the habitual and the continuous interpretation (see the next section), is acquired at the age of approximately 5–6;5 [24–25]. Verbal aspect lies in different interfaces, i.e., internal (morpho-syntax) and external (semantics). The mapping of the morphological features of perfective correspond to one semantic interpretation while the morphological form of imperfective corresponds to two interpretations. It has been observed that heritage speakers are prone to morphological loss and simplification of verbal forms by re-organizing the verbal structures [26,27]. Scontras et al. [28] support the restructuring of micro- and macrostructures by heritage speakers, who favor simplicity over complexity of particular structures and morphological features. What is expected for Greek heritage speakers is to pattern like other groups of heritage speakers, i.e., Russian, facing difficulties with the fused morphology and the adjustments needed to form the perfective aspect. In addition to that, if the mapping of imperfective interpretations has not been acquired during the critical age of approximately 5–6;5 according to acquisition studies (cf [29] and the Timing Hypothesis for an overview of different phenomena among monolingual and bilingual speakers), then heritage speakers are expected to show inconsistencies regarding this late-acquired phenomenon.

### Greek verbal morphology

Greek is a highly inflected language with rich morphology (cf. [30]:124 for an overview). Verbal morphology in Greek is fusional, meaning that aspect, voice, tense, and subject-verb agreement are marked on the verb as it has been proposed by Philippaki-Warburton ([31]:161) for the non-imperative verb forms seen in (1).

**Table:** 

(1)	Infl [Agr+Tense] Voice Aspect [VP V]

**Fig 1:**
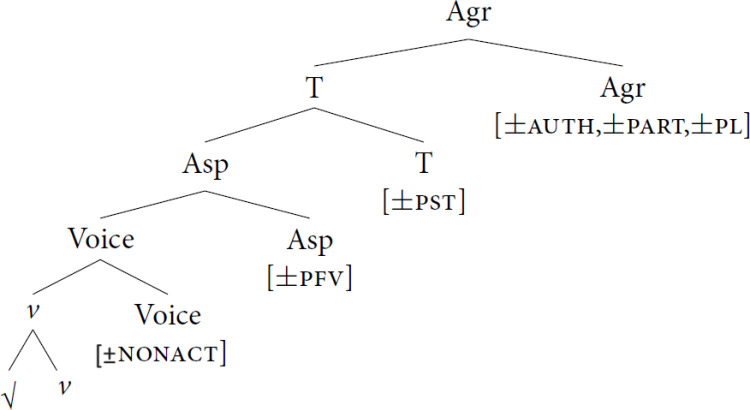
The verbal complex.

The syntactic representation of the verbal complex can be seen in Fig 1, taken from Paparounas [32]. According to Ralli [33], mood has lost its overt marking in Modern Greek, while Philippaki-Warburton [31] claims that only imperative forms are marked on the verb. Subjunctive mood is marked on particles [34]. In this study, emerging patterns in the suffixal morphology are explored by looking primarily into verbal aspect, which is in interdependence with the verbal complex.

Besides the verbal features mentioned in Fig 1., another element is added at the beginning of the root to facilitate the stress patterns. The vocalic element/e/ is called augment and is connected with the presence of stress and more precisely when the antepenultimate syllable stress law causes a left-hand stress shift outside the confines of the word while it is absent in unstressed position [30,35]. This vowel could also be a potential past tense exponent alongside the stress shift [31:161]. The augment is realized in all 3 persons of singular in past tenses to facilitate the stress in the penultimate syllable. According to Tsolakidis et al. [36] Greeks of 1st generation immigrants who emigrated in Canada (age range 42–73 years old) originating from different places of Greece and having low educational background exhibit an inconsistency of the non-stressed augment which is considered to be a characteristic of the vernacular intertwined with dialectal elements.

#### Greek verbal morphology in the 1st Conjugation Class.

In Greek, there are two major Conjugation Classes (CC) under which the verbs exhibit inflectional patterns. There is also a 3rd CC according to Holton et al. [37] and Spyropoulos et al. [38] consisting of 7 specific verbs which possess a null morpheme meaning that they do not have an overt verbalizing suffix, e.g., akú- Ø-o ‘I hear’. These verbs are not included in the experimental items. In the 1st CC can be categorized the verbs in which stress falls on the root of all IPFV [-PAST] forms like gràf-o ‘write’.

After Ralli’s [30,35] analysis on verbal inflection depending on the non-/existence of stem allomorphy, the latest morphological theory for verbal analysis in Greek has been proposed by Spyropoulos et al. [39] and Revithiadou et al. [40] couched within the “Distributed Morphology” model. According to those scholars, the verbal root combines with overt or zero exponents to realize the features that are encoded in every verb in Greek, as seen below (examples 2–5). There are three main categories under which the verbs are classified.

Firstly, there are the regular/strong verbs in which the morpho-phonological rules do not affect the segments of the morphological features. In this classification, there are two subcategories that separate the verbs in those with vowel ending roots (2a-b) and consonant ending roots (2c-d). In 2d, a morpho-phonological rule operates and the fricative ‘f’ turns into ‘p’ because of manner assimilation. Regarding the realization of the aspect, it is important to identify the phonological profile of the root. The roots of strong verbs are fully specified and are selected by the fully specified affixal exponent/s/, which indicates the [+PFV] value. In order to keep the tense value consistent in all examples, namely [−PST], the [+PFV] value appears in subjunctive mood indicated with the particle na ‘to’ unlike the ΙPFV value, which appears in the indicative mood.

**Table:** 

(2)	a.	iδri-	Ø	Ø			-o	
		root-	verbalizer-	voice/aspect			-tense/agr.	
		establish.ACT/IPFV.[-P ST]/ 1SG						
	b.	na	iδri-	Ø	-s			-o
		SBJV	root-	verbalizer-	voice/aspect			-tense/agr.
		establish.ACT/PFV.[−PST] / 1SG						
	c.	γráf-	Ø			Ø		-o
		root-						verbalizer-						voice/aspect												tense-agr.
		write.ACT/IPFV.[-P ST]/ 1SG						
	d.	na	gráp	-Ø	-s	-o		
		SBJV	root-			verbalizer-			voice/aspect-			tense-agr.		
		write.ACT/PFV.[-PSΤ]/1SG						

As strong verbs are classified also some verbs that their stem end in a coronal/n/ and have a phonologically predicted alternation as in (3a-b). In this category there is an inconsistency for verbs that end with the coronal/n/ as not all verbs have a phonological predicted alternation with/s/ as happens with the verb molìn-o ‘pollute’.

**Table:** 

(3)	a.	lìn-o	
		solve.IPFV.[−PST]/1SG.	
	b.	na	lì-s-o
		SBJV	solve.PFV.[−PST]S/1SG.

The second classification concerns the irregular/ weak verbs in which ‘the realization of certain functional categories affects the phonological shape of the root’ [40]. The roots undergo reshaping due to phonological adjustments, suppletion, and omission or addition of a consonant and syllables (cf. [39,40]). Based on Spyropoulos et al. [39,41] and Revithiadou et al. [40], verbs that fall under the categorization of root allomorphy or stem alternation exhibit systematic patterns of vowel (4a-b) and consonant alternation (4c-d) which are analyzed according to the notion of “Gradient Symbolic Representations” and are subject to readjustments applied on a single root rather than on different roots and allomorphic stems. As the scholars mention, in example (4a), the root vowel/e/ followed by the coronal/n/ compose the imperfective form while the/n/ disappears and the root vowel alters to/i/ to transfuse the perfective value.

In (4c-d), there is a consonant absent in the other environments but imperfective, and the coronal sound/n/ in (4c) that is produced leads to the association of this specific position with aspect. Furthermore, in example (4c), a morpho-phonological rule applies in the consonant/x/, which turns it into/k/ in (4d) due to voice and manner assimilation. All these alternations are morpho-phonologically conditioned.

**Table:** 

(4)	a.	stèln-o	
		root-agr.	
		send. ACT/IPFV.[-PTS]/1SG	
	b.	na	stíl-o
		SBJV	root-agr.
		send.ACT/PFV.[-PSΤ]/1SG	
	c.	spróhn	-o
		root-	agr.
		push.ACT/IPFV.[-PST]/1SG	
	d.	na	sprók-s-o
		SBJV	root-agr.
		push.ACT/PFV.[−PST]/1SG	

Finally, one more classification of verbs is based on strong suppletion [40], under which verbs undergo a complete stem change as in example (5).

**Table:** 

(5)	a.	trò-o	
		eat. IPFV.[−PST]/1SG.	
	b.	na	fà-o
		SBJV	eat. PFV.[−PST]/1SG

#### Greek Verbal Morphology in 2nd Conjugation Class.

In the 2nd CC are categorized all verbs that exhibit non-root stress for example agap-άo/ό ‘love’, poth-ό ‘desire’ [42]. According to Ralli [30,35] these are the verbs that rely on the variation (Y⁓Yi) like in example (6) and have two allomorphic stems.

**Table:** 

(6)	Systematic allomorphy	2 allomorphic stems	pul(à)-o	na	pulì-s-o
			sell.IPFV.PRS.1SG	SBJV	sell.PFV.[−PST]/1SG

According to Spyropoulos et al. [38], the verbs of the 2nd CC take a vocalic extension, which is abstract between the root and the inflectional formatives. This vocalic extension functions as a verbalizer v which either remains empty (example 7a) or is materialized with a vocalic element (7b-c). The absence or the presence of the vocalic element plays a significant role in the facilitation of stress. In the case of 2nd CC verbs, the root is categorized by this vocalic element that functions as a verbalizer and indicates the grammatical aspect of the verb form. The verbalizer/u/ in example (7b) denotes the imperfective aspect, while the verbalizer/i/ denotes the perfective aspect in example (7c).

**Table:** 

(7)	a.	aγap-Ø -o
		love.IPFV.VRB.[−PST]/1SG.
	b.	aγap-ú-s-a
		love.IPFV.VRB.[+PST]/1SG.
	c.	aγap-í-s-a
		love.PFV.VRB.[+PST]/1SG.

Typical for southern Greece is the alternation of the vocalic element to/a/ and the addition of one more consonant -γ- between the root and the inflectional ending (example 8), which is used mainly in the oral speech, and even extended in parts of Thessaly (central Greece) and Epirous (north-western Greece) [42,43]. In their study, Tsolakidis et al. [43] found that the existence of -aγ- is even evident in the productions of participants that originate from northern Greece, and in general, participants switch between the two types. The type in example (8) is used interchangeably with the one in example (7b).

**Table:** 

(8)	a	γàp-a-γ-a
		love.IPFV.VRB.[+PST]/1SG.

## Aspect in English and German

Grammatical aspect in English is associated with Simple Past and Past Progressive, the former encodes perfectivity and the latter either continuity or progressivity (Smith [17]). In English, the verb is marked with the progressive imperfective **-**ing suffix, while tense is indicated on the auxiliary verb *be*. In Simple Past, the **-**ed ending added to regular verbs can convey meanings of perfectivity or even habituality. Habituality can also be expressed through periphrastic constructions such as *used* *to* and *would*.

German does not mark grammatical aspect on the verb. Habituality and continuity can be indicated using periphrastic constructions. Habituality, in particular, can be expressed in both the present and past tenses through phrases like *pflegen zu* ‘used to’ and d*ie*, *Gewohnheit haben zu* ‘have the habit of’ followed by infinitives (Löbner [44]). In past tenses, habituality can also be conveyed using the Preterite. Continuity, on the other hand, is expressed through the temporal adverb *gerade* ‘now’ or through two types of periphrastic constructions: *am/beim* ‘on/at’ followed by an infinitive, and *dabei sein zu* ‘be about to/in the process of’ followed by an infinitive (Sioupi [45]).

## Previous studies on verbal morphology and heritage speakers

Numerous scholars investigating heritage grammars highlight challenges heritage speakers face, particularly in the verbal domain. Research indicates that these bilingual individuals often experience difficulties with morphological features, leading to simplifying inflectional elements and divergence from standard verbal paradigms (cf. [46]: for an overview). One of the most influential theories is the ‘Bottleneck Hypothesis’, which suggests that learners may successfully acquire syntax, semantics and pragmatics but the functional morphology is the key constraint in acquisition [47].The Bottleneck Hypothesis is based on the idea that functional morphology is crucial because it captures the syntactic and semantic distinctions between languages. While core syntactic operations and the calculation of meaning are universal, learners must navigate through functional morphology to acquire syntax and meaning in a second language. As a result, morphology becomes the primary constraint in the process of language acquisition. The ‘Bottleneck Hypothesis’ applies also to heritage speakers, as proposed by Montrul [48] and Mikhaylova [49]. Moreover, this hypothesis is particularly relevant for languages with rich morphology, as is the case of languages like Russian, Spanish etc., and in the present paper Greek. According to Mikhaylova [50], heritage speakers exhibit sensitivity to aspectual contrasts, i.e., to boundedness of events, in a similar way monolinguals do. On the other hand, this resemblance to monolingually-raised groups seems to be selective, as their performance on lexical aspect and more precisely on telicity and the related morphological features patterns with that of L2 learners. What Scontras et al. [28] observed, is a reorganization of morphosyntactic features in microstructures and a reanalysis of arguments in macrostructures, leading to a simplification of heritage speakers’ grammars. Thus, Scontras et al. [28,51] proposed the “Representational economy” which leads to a reduction and reanalysis of heritage speakers’ grammars, impacting the production of novel trajectories. Heritage speakers tend to simplify morphologically complex structures by applying the default or the most uncomplicated and transparent in every instance feature.

Much research has been conducted on heritage Russian in the US. By profiling Russian heritage speakers, Polinsky [52] revealed discrepancies in verbal morphology, and these challenges arise due to Russian being a highly inflected language, akin to Greek. The verbal paradigms decline in heritage grammar in conjunction with the lack of S/V agreement and the absence of subjunctive mood. Heritage speakers tend to retain verbal items based on the frequency of input, and only proficient speakers utilize past tense forms. In American Russian, aspectual pairs are often replaced by lexicalized perfectives for inherently telic verbs and imperfectives for inherently atelic verbs. Additionally, some lexicalized verbs are substituted with periphrases, indicating an avoidance strategy favoring non-inflected forms. In her book, Polinsky [6] cites several studies and examples of heritage speakers, emphasizing English heritage speakers as a prime example. Those lacking salient input tend to overapply the/-en/ ending in irregular verbs to create the participial form or overuse the/-ed/ marker, leading to regularization patterns ([6]: 165,173).

Overregularization patterns in Spanish present tense morphology are found among Spanish heritage children having American English as their dominant language as well [53]. In written tasks, children exhibited difficulties with verb stem alternations in present tense compared to monolingual children. Spanish is a language with rich inflection, like Greek, with 3 main CC and several features like tense and aspect marked on the verb. Heritage Spanish children tend to simplify and regularise the verbs that require stem alternation, while the irregular verbal morphology remains largely intact. Another study by Fernández-Dobao and Herschensohn [54] reveals that heritage children commonly overregularize stem vowels in production tasks, attributing it to a typical developmental error.

A recent study by Uygun et al. [55] reveals that Turkish heritage speakers overapply the regular morphology in nonce verbs and they prefer the generalization of the regular Aorist as this is the default strategy to mark the past tense compared to morphologically irregular verbs. This preference of rule-based verb formation might indicate that heritage speakers face difficulties with the inflectional morphology albeit regular marking of the Turkish Aorist is the most frequent strategy.

Studies on heritage Icelandic in Canada examine the tense morphology and verbal aspect. A study by Jóhannsdóttir [56] reveals that the progressive is used more frequently in heritage Icelandic compared to homeland Icelandic, signalling to an overstandardization of the verbal morphology as more features and inflectional elements are needed to form the simple present and the past. In terms of lexical aspect, heritage speakers use temporal adverbials more often to indicate the notion of simultaneity in heritage Icelandic compared to their majority language, namely English [57]. The structure of the progressive aspect in Icelandic is formed differently from English and thus, speakers differentiate their narrations and are overexplicit while expressing the simultaneous interpretation.

Delving into processing studies, Romanova [58] explored the processing mechanism of verbal morphology in non-past tense on native speakers, heritage speakers and L2 learners of Russian testing those groups both with real and nonce verbs from almost all verb classes. Heritage adolescents and young adults exhibit, on the one hand, stem generalizations and regularization patterns in real verbs but on the other hand, they exhibit a divergent attainment in nonce verbs resembling the processing of L2 learners signalling to class levelling. The researcher states that formal literacy in heritage Russian is a factor that affects their performance in verbal morphology and increases grammatical competence.

There are also studies which support that heritage speakers have acquired the verbal inflectional paradigm, such as Flores et al [59]. Through narration tasks, they tested the verbal morphology in heritage Portuguese children and adolescents who reside in the German-speaking part of Switzerland in both their languages. The mean number of different verb types that every child uses in both languages is identical, exhibiting robust knowledge of verbal morphology. Although there are a few overregularization patterns, i.e., in irregular past tense morphology in their dominant language, which are rated lower than 4%, the speakers are quite competent in this domain. Furthermore, regarding the aspectual patterns the researchers observed an infelicitous use of imperfect forms in Portuguese at a rate below 7% and in German below 2%. As the authors state, “morphological and syntactic deviations are residual in both languages,” but these bilinguals “display stable syntactic and morphological knowledge in both their languages.”

## Acquisition of aspect in Greek as a heritage language and as L1

Aspect is regarded as a highly susceptible phenomenon in the context of language contact, particularly in languages that possess fusional morphology, such as Greek. The pioneer in reporting the difficulties in different domains of the Greek grammar and pointing to a deviation from the monolingual norm is Seaman [60]. He profiled Greek heritage speakers in the US, reporting that these speakers tend to simplify and reduce the complexity of the Greek verbal system ([60]: 164). Firstly, he mentions that heritage speakers prefer to use verbs without morphological distinction regarding aspect as it is the light verb kano ‘do’. Alexiadou and Rizou [61] corroborate this by showing a clear preference for periphrastic constructions with light verbs instead of lexical verbs with prefixes and stem alternations. Evidence on analytic forms comes from Boon [26] who reports that Welsh heritage speakers prefer to resort to periphrastic constructions with the auxiliary “to be” over synthetic forms as Welsh is also a language with fusional morphology. Furthermore, Seaman ([60]: 146-7) observes overcorrections regarding the aspectual marking of verbs in spoken data of Greek heritage speakers in the US and finally, he reports simplifications in the voice system illustrating a deponent verb exhibiting [−NAct] voice while it should exhibit [+NAct] voice in standard Greek. In line with this finding is an upcoming study by Alexiadou and Rizou [62], which presents novel patterns in voice alternation in heritage speakers’ grammars.

A further study on adult Greek heritage speakers was conducted by Paspali et al. [63]. The researchers checked the accuracy of Greek heritage speakers residing in the US and Germany via a speeded grammaticality judgment task. The German group performed monolingual-like, while the group in the US, although it was the least accurate one, scored significantly better in the imperfective conditions than in the perfective ones, indicating that these speakers prefer the morphologically unmarked type and specifically the habitual one. In the same study, Paspali, Rizou and Alexiadou [63] mention a few patterns of novel verbal forms produced by Greek heritage speakers, which are elaborated on thoroughly in the present study.

Focusing on Greek L1 children, Dosi [64] performed an elicited production task, the same task performed in the present study (see Materials and methods), reporting the difficulties that heritage speakers faced with the morphological forms of aspect. She focused, though, only on the accuracy rates of her groups without discussing any morphologically novel forms.

Different scholars performed processing studies on Greek monolingually-raised speakers investigating whether and how the morphological feature of aspect on the verb stem and/or the CC affect participants’ performance. Tsapkini et al. [65] cautiously stated that verbs with allomorphic stems show the same facilitation as regular verbs do, and there is no differential priming elicited for any of the morphological categories besides those which entail a vocalic allomorph, but still, this remains unclear as confounding factors persist, while the results on plural morphology in nouns are more transparent. In corroboration to this, Soukalopoulou [66] suggests that the distinction between the sigmatic and asigmatic morphological marking does not capture the verbal complexities of the Greek [+PFV] aspect. Based on the ‘Dual-Mechanism Model’ she proposes that verbs with predictable stem alternations and the PFV marker/-s/ are considered to have regular and sigmatic stems because these are computed by rule-based operations, while the irregular and asigmatic verbs have non-predictable stem alternations and are stored as full forms in memory. Elaborating further on verbal morphology and including more parameters Bompolas et al. [67] related the length of the lexical item with the acquisition of verbal morphology. Short irregular verbs in Greek are acquired earlier than length-matched regulars of the same frequency, while long irregulars are acquired later compared to all other forms. In general, morphologically fully transparent past-formation stems are processed and acquired more easily compared to non-transparent and opaque forms because the latter stems have to undergo several processes of stem alternation. The researchers highlight that the type of stem allomorphy itself matters.

Having reviewed several studies concerning different heritage speaker groups and individuals raised exclusively in a Greek-speaking environment, we now advance to formulating three research questions (RQs). The goal is to gain insight into the morphology of verbal constructions in heritage speakers’ grammars.

## Research questions

Due to the difficulties that heritage speakers face with verbal morphology based on previous literature, this study addresses 4 RQs based on the relevant hypotheses (H) and formulating the predictions (P) regarding the production of morphologically novel forms.

RQ1: Given the rich verbal morphology in Greek, do heritage speakers exhibit non-canonical patterns in the domain of aspect, deviating from monolingually-raised speakers’ productions? If so, do heritage speaker groups differ significantly from the monolingually-raised group in terms of the production of non-canonical forms?

H1: Based on the Bottleneck hypothesis [47], extended to heritage speakers [48–50], the more features and the more morpho-phonological rules that a verbal stem encodes, the more susceptible to change the structure becomes. As Mikhaylova [49] supports, heritage speakers of Russian exhibit discrepancies with aspectual morphology, leading to a decisive hindrance in comprehending the aspectual distinctions.

P1: We expect heritage speakers to produce emerging, novel, and non-canonical patterns compared to monolingually-raised speakers of Greek, given the morpho-phonological complexity in marking the [+ PVF] aspect, as shown in examples (2b, 2d, 3b, 4b, 4d, 5b, 7c) among other features in the Greek verbal system.

RQ2: Do heritage speaker groups differ significantly from each other regarding the production of novel morphological forms? This question aims to identify whether the two heritage speaker groups recruited in the US and Germany differ statistically from each other in terms of the production of non-canonical forms that encode untargeted features. Do they exhibit mismatches in features that a verb encodes, namely tense, voice, morphophonological alternations like vocalic elements, stress pattern, φ-features (person and number) in combination with the aspectual feature?

H2: Having in mind the majority language of the heritage speakers, it is known that English has poor agreement while other Germanic languages like German have rich agreement, especially in their verbal system [68–69]. Based on the fact that verbs in German encode different features unlike English and on the hypothesis proposed by Scontras et al. [28] concerning the representational economy, a different performance between Greek heritage speakers in Germany and in the US is expected. The latter Hypothesis by Scontras et al. [28] proposes a reorganization of the verbal morphosyntactic system, leading heritage speaker grammars to a divergent attainment by simplifying complex morpho-phonemic rules.

P2: We expect Greek heritage speakers in Germany to produce fewer novel verbal forms compared to heritage speakers in the US firstly based on the fact that German is a morphologically richer language compared to English and secondly based on their performance on aspect in the study by Paspali et al. [63]. Furthermore, heritage speakers are expected to simplify the verbal forms and produce in case of aspect the unmarked forms as shown in examples (2a, 2d, 3a, 4a, 4c, 5a, 7b, 7d), while in the case of tense the forms that indicate the present tense and avoidance of the augment.

RQ3: If indeed novel verbal forms are detected, could any patterns of interference from the majority languages be identified in these novel forms?

H3: Within the bilingual speaker’s lifespan, transfer effects from the dominant language are observed to compensate the gaps in their heritage language caused by the limited exposure and input [70,71]. According to Polinsky [6] morphology weakens in heritage grammars, and this fact might facilitate the interference from the majority language. In combination to H2, German is a rich agreement language like Greek, but the same does not hold for English. The rich verbal system of German and the poor verbal system of English might differently affect the performance on Greek.

P3: We expect that patterns signaling interference will be detected either in the Greek heritage group recruited in Germany or in the US. There might be a possible hindrance in the different mapping of the verbal features that the majority and the heritage language encode.

RQ4: If the two heritage speaker groups differ significantly from each other, which are the factors compiling the speakers linguistic profile that might correlate and be predictors for improvement of fully inflected verbs in the appropriate environments? This RQ is based on several language background variables that are collected in a form of a questionnaire profiling the heritage speakers.

H4: The profile of the heritage speakers, meaning the data compiling the speakers’ linguistic profile, play an important role in the performance in the heritage language [8–12].

P4: We expect that the two groups differ in terms of their language background data. Most importantly, we predict that the formal education received in the heritage language, as confirmed in the study by Paspali et al. [63], would be a significant predictor in the present study as well, given the fact that the two countries follow different curricula regarding bilingual education.

## Materials and methods

In this section, the task design and the profile of the participants are presented in detail. The following study was conducted according to the guidelines of the Declaration of Helsinki, and approved by the Ethics Committee of the Deutsche Gesellschaft für Sprachwissenschaft (German Linguistics Association). Date of Approval: 20 November 2017. This study does not constitute a dual publication and only a part of it is published in the peer-reviewed paper by Paspali et al. [63]. In the present study an error analysis of the novel forms is conducted, while in Paspali’s et al. [63] study the focus is on the accuracy rates among the three groups (heritage speakers in Germany, heritage speakers in the US and Greek monolingually-raised speakers).

### Elicited production Task

To elicit verbal forms marked for grammatical aspect an oral elicited production task in the form of sentence completion was used. The task was created by Agathopoulou and Papadopoulou [72] and performed in many studies thereafter [63,64,73,74]. Dosi [64] has made a few changes in the experimental items, which were modified further in Rizou’s [74] and Paspali’s et al. [63] studies. The purpose of the task is to investigate only the verbal feature of grammatical aspect in controlled environments by keeping all other verbal features parallel such as past tense, active voice, indicative mood and 3^rd^ person either singular or plural. The full list of the experimental items can be seen in S1 Appendix. All targeted verbs appeared at the end of each sentence in brackets in the 1st singular person so the informants were able to process the conjugation derivation. In order to avoid any ambiguity because Greek is a pro-drop language, the subject was indicated so participants could mark the appropriate person feature in the verb suffix. Finally, in a few sentences, a temporal adverbial phrase was added to denote clearly a reference to the past.

This specific task consists of thirty items in total mapping the three conditions of grammatical aspect, namely 10 sentences denoting the perfective aspect, 10 denoting the imperfective continuous interpretation and 10 the imperfective habitual, respectively. 2 pilot sentences proceded the 30 experimental items so participants could get acquainted with the procedure. There were no idiomatic expressions with figurative meaning in order for the sentences to be more semantically transparent to the bilinguals.

As seen in Table 1, there are 10 strong verbs that require an exponent/s/ to mark the aspectual feature, i.e., ràvo - érapsa ‘sew- sewed’, 9 verbs that exhibit weak allomorphy requiring the exponent/s/, and 4 verbs that exhibit only weak allomorphy and vowel alternation patterns. Finally, there is 1 verb with strong suppletion, although marginally, which requires root allomorphy, i.e., piγèno - píγa ‘go- went’. 21 out of thirty verbs belong to the 1st CC, and 9 of them to the 2nd CC. In Table 1 are seen only 17 verbs in the 1st CC and the reason for this is because the verbs dulèvo ‘work’, malòno ‘fight’, γirízo ‘come back’ and stèlno ‘send’ appeared in 2 experimental items each resulting in 21 experimental items. The verb ponào ‘hurt’ of the 2nd CC appeared in 2 the experimental items as well.

**Table 1:** Distribution of verbs per morphological formation and conjugation class.

	Strong verbs consonant ending	Weak allomorphy + s	Weak allomorphy	Strong suppletion
1st Conjugation Class	ràv-o	δiòhn-o	stèln-o	piγèn-o
	lìn-o	spròhn-o	plèn-o	
	psìn-o		pèrn-o	
	hàn-o		fèrn-o	
	malòn-o			
	vàf-o			
	horèv-o			
	γràf-o			
	δulèv-o			
	γiríz-o			
2nd Conjugation Class		pulà-o		
		pernà-o		
		kolà-o		
		forà-o		
		ponà-o		
		milà-o		
		γelà-o		

Table 1 illustrates the categories of the verbs, that were tested in the elicited production task, according to the classification proposed by Spyropoulos [39] and Revithiadou et al. [40]. More precisely, the table presents the morphological rules applied to the verbs in order to form the perfective aspect. There is a further distinction of the verbs according to the CC they belong to.

The verbs are selected carefully as none of the them consists of a loan stem and the verbalizer/-ar/ like park-ar-o ‘park’. No prefixed verbs are included either. The former verbs, namely the ones with the verbaliser/-ar/ do not exhibit any morphological distinction in [+/−PFV] marking [75–76] while the latter, the prefixed ones required further morpho-phonoligical alternations in past tenses and their meaning can vary depending on the prefix [33].

The task was performed orally. The elicitor read the sentences out loud, doing a 2-second pause in the place where the verb should be produced. Participants had to repeat the whole sentence and fill in the gap with the appropriate verbal form. The participant’s responses were being recorded. The oral mode was chosen because of the participants’ different literacy levels, as not all of them systematically attend or have attended courses in their heritage language. The instructions the elicitor gave were: “I will read the sentences out loud doing a small pause in the blanks and you will fill those blanks with the verb in the brackets. Of course, you are allowed to look at the sentences while I am reading them, and if you don’t listen clearly something, I can repeat the sentence. I am allowed to read each sentence twice if needed. The following events happened in the past.” In case participants produced the present tense of the targeted verb, the elicitor asked him/her: “How can we describe this action, if this happened in the past?”. If the informant insisted on his/her answer, then this was the final answer that was coded.

### Participants

Table 2 presents the participants’ pool. The speakers are 189 in total, distributed in the three groups, heritage speakers in Germany, in the US and the monolingually-raised group which is called control group. Participants were recruited in urban areas; heritage speakers in Germany were recruited in Berlin, heritage speakers in the US in New York, NY and Chicago, IL metropolitan areas; and finally, monolingually-raised participants were recruited in Athens, Greece. Participants in the US were tested from October 10th, 2018 until March 20th, 2019, in Berlin from September 1st, 2018 until January 30th, 2020 and in Athens from March 10th until March 30th 2019. All participants, or parents/guardians of underage participants signed a consent form in accordance with the Declaration of Helsinki in their respective majority language, meaning that bilingual participants in the US read and signed the consent form in English, participants in Germany in German and monolingually-raised participants in Greece in Greek. Furthermore, they assured the elicitors that they had normal or corrected-to-normal hearing and vision, and had no speech disorder. Bilingual participants performed some tasks in both their heritage and their majority language with the supervision and guidance of a native speakers in the respective language. As stated in the consent form, participants’ identities were anonymized by attributing them a unique code, as seen in example (10) in the Results Section and onwards. The first two capital letters identify the country from where each participant was recruited, i.e., US stands for USA, DE for Germany, and GR for Greece. The following two letters indicate whether the participant is monolingually-raised (mo) or bilingual speaker (bi). Then, a unique 2-digit number is given to each participant starting from 01 to 99. The next letter reveals the gender of each participant. For example, F stands for female and M for masculine participants. There were no cases of diverse gender. Finally, the last letter, in the case of participants in this study is G meaning that the language, in which they performed the experimental tasks was Greek.

**Table 2:** Participants’ data that compile their linguistic profile across groups.

	HSs in Germany	HSs in the US	Control Group
N	48	77	64
	(24 Females)	(44 Females)	(32 Females)
Mean Chronological Age	23;2	22;1	21;5
	SD 6.900	SD 7.460	SD 6.638
	(min 14 – max 36)	(min 14 – max 35)	(min 13 – max 35)
Mean Age of Onset	1;9	1;4	–
	SD 2.184	SD 1.995	
	(min 0 – max 8)	(min 0 – max 6)	
Hours of education in Greek (HL)	5984	2095	–
	SD 4708.910	SD 856.429	
	(min 0 – max 12480)	(min 0 – max 3120)	
Years of education in Greek (HL)	7;0	9;1	–
	SD 3.929	SD 3.339	
	(min 0 – max 12)	(min 0 – max 12)	
Self-ratings in Greek (HL)	4,05	3,74	4,87
	SD.8604	SD.9140	SD.3118
	(min 2,25 – max 5)	(min 1,75 – max 5)	(min 3,25 – max 5)
Literacy practices	1,25	1,03	1,54
	SD.5768	SD.5135	SD.4614
	(min 0 – max 2)	(min 0 – max 2)	(min 0,3 – max 2)
Current input in Greek (HL)	1,11	0,81	–
	SD.4655	SD.3236	
	(min 0 – max 1.83)	(min 0 – max 1.50)	

Right after the data collection in February 2020, all speakers were anonymized according to the guidelines provided above and the data compiling the linguistic profile of the speakers were assigned to each participant respectively. Access to the non-anonymized data had only the first author of the present study, while full access to the anonymized data had the two co-authors of the present study, and partial access had the first author in [63] publication (see the acknowledgements section).

The data compiling the speakers’ linguistic profile, such as the mean chronological age and the age of onset to bilingualism and specifically to the majority language, were collected by means of a questionnaire at the end of the elicitation task and are illustrated in Table 2. The cumulative exposure to formal bilingual education was measured both in years and in hours of Greek lessons every bilingual participant attended. While the US group exceeds the German group in years of education, the latter surpasses the former in hours of education. This can be explained by the different curricula in Germany and in the US. In Germany, heritage speakers of Greek usually attend bilingual schools, while in the US they attend weekend or afternoon school provided by the Greek Orthodox church. The self-reports on Greek proficiency consisted of 4 questions on listening, reading, writing, and comprehension of the Greek language on a 1–5 scale for each question. An average score was calculated for each group. An average of literacy practices was also calculated for each participant both for his/her heritage and majority language (for the monolingually-raised group, only one average score was computed). Scores were calculated based on 3 questions with respect to the frequency of the input they receive from media and their written production in the different means of communication on a three-point scale (often/ sometimes/ never). An average of current input was also calculated on a three-point scale (every day/ a few times per week/ a few times per month). Participants had to indicate which language they speak to core members of their family and friends and which language is also spoken to them. The latter variable is not computed for the control group because monolingually-raised speakers address their family members and friends in Greek and consequently these members address them back in Greek as well.

### Data coding

The data collected from the first author and well-trained research assistants were transcribed by the first author, and they were coded and annotated by the first and second authors, ensuring interrater reliability. As the focus of this manuscript is the alternative forms, data exhibiting only the non-targeted aspectual features (example 9a) are beyond the scope of the study, while verbal forms that exhibit either a non-targeted verbal feature beside aspect or in addition to aspect are the focal point of the paper (example 9b).

**Table:** 

(9)	a.	Xthes	oli	mera	*ponese	to	kefali	mu.	
		Yesterday	all	day	hurt.PFV.PST	the	head	my.	
		Yesterday my head was hurting all day long.							
	b.	Xthes	oli	mera	*ponase/	*ponai	to	kefali	mu.
		Yesterday	all	day	hurt.?IPFV/PFV/	hurt.IPFV.PRS-3SG	the	head	my.
		Yesterday my head was hurting all day long.							

The first categorization is made based on the existence of the verbal form, whether this form is grammatical in any other tense, voice or mood or it is novel. A further categorization based on the erroneous feature/s produced is applied for the morphologically-existing forms. One point is given for every non-target verbal feature, i.e., aspect, tense, voice, number, person and mood. For the morphologically non-existing ones the categorization is made based on common patterns detected in the forms such as stress misplacement, CC change, erroneous augment, absence or presence of the/s/ exponent for the relevant forms, stem change, non-past suffixation. In this case, the boundaries between the patterns are not distinct and thus, the scoring system might overlap between the patterns, meaning that not only one pattern applies to each verbal form.

## Results

This section provides the results of heritage speakers’ performance on the production task quantitatively followed by an extensive qualitative analysis of the deviant responses including the morphologically novel structures.

As mentioned in the previous subsection, the alternative forms are categorized based on their morphology in morphologically existing and non-existing verbal forms. The former category includes examples (10–16) which are illustrated below, while the latter category includes examples (17–28).

**Fig 2:**
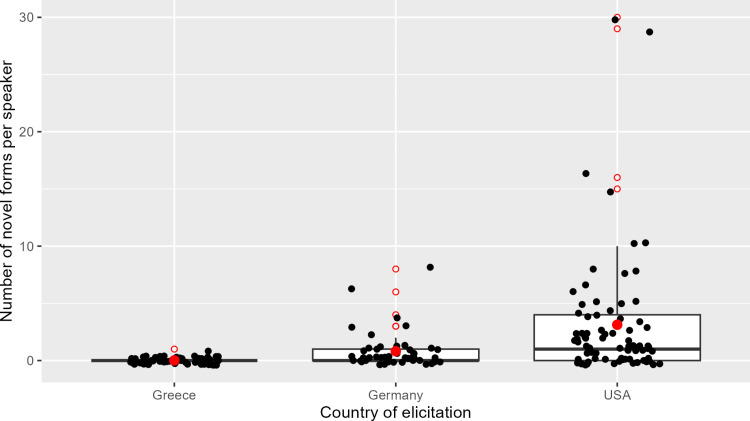
Distribution of novel forms by country and speaker.

For a general overview of the data, we turn to Fig 2 which shows the distribution of novel forms including both the morphologically existing and the non-existing ones, per group of speakers. The speakers’ groups on the x-axis are represented according to the country of elicitation and the y-axis shows the amount of novel forms by participant. The mean is marked by a red dot and a median marked by a black horizontal line. Each black dot represents one individual speaker, while the empty red circles show outliers regarding the underlying distribution in the boxplots.

A generalized linear mixed model (GLMM) was employed to analyze the relationship between the number of novel forms used (N) and the country of participants, incorporating participants as a random effect. The model was fitted using a Poisson distribution with a log link function [77]. The Poisson Distribution is favored for count data, like number of novel forms per participant, because the average rate of occurrences, especially in the group of heritage speakers in Germany is small and thus the Poisson Distribution provides a good estimate of the probability of the data points. The model accounted for 189 observations, each corresponding to a unique participant, resulting in 185 residual degrees of freedom. In terms of random effects, the model estimated a variance of 1.597 for the participant random intercept, with a standard deviation of 1.264. This suggests variability in the baseline use (monolingually-raised group) of novel forms across participants, which was accounted for in the model.

The fixed effects of the model revealed significant differences between the countries. The intercept, representing the baseline use of novel forms for the reference category ‘Greece’, was estimated at -4.774 with a highly significant p-value (p <.001). This indicates that the baseline use of novel forms for the monolingually-raised participants from Greece is relatively low. The coefficient for ‘CountryGermany’ was estimated at 3.760 with a significant p-value (p <.006), suggesting that participants from Germany demonstrate a significantly higher use of novel forms compared to those from Greece. Similarly, the coefficient for ‘CountryUSA’ was estimated at 5.118, again with a significant p-value (p <.001), indicating an even higher use of novel forms for participants from the USA relative to those from Greece. The correlation between the fixed effects showed a strong negative correlation between the intercept and the country coefficients, reflecting dependencies between these factors.

Further analysis using Tukey’s post hoc test demonstrated significant differences between all pairs of countries. The difference in the use of novel forms between Germany and Greece was estimated at 3.760, with a p-value of 0.014. The difference between the USA and Greece was even more pronounced (p <.001), with an estimate of 5.118. Additionally, the comparison between the USA and Germany yielded a difference of 1.357, with a highly significant p-value (p <.001). These results indicate that participants from the USA had the highest use of novel forms, followed by those from Germany, and finally those from Greece, with statistically significant differences between each pair.

The regression table below (Table 3) summarizes these findings, providing estimates, standard errors, z-values, and p-values for both the fixed effects and the Tukey’s post hoc comparisons. The asterisks symbolize the levels of statistical significance, underscoring the robust nature of the results.

**Table 3:** Generalized Linear Mixed Model Results for the Use of Novel Forms Across Different Countries.

FixedEffects	Estimate	Std.Error	z value	p-value
Intercept	−4.774	1.342	−3.556	0.000***
Country: Germany	3.760	1.373	2.740	0.006**
Country: USA	5.118	1.354	3.780	0.000***

Table 3 presents the estimated coefficients, standard errors, z-values, and p-values for the fixed effects of country and the random effects for participants. Additionally, Tukey’s post hoc comparisons between countries are included to highlight significant differences in the use of novel forms.

Table 4 presents the raw numbers and the percentages of the alternative forms distributed in the two heritage speaker groups. The percentages are computed horizontally, exhibiting the raw number, the speakers who produced these forms and the percentages of the total alternative forms, then the morphologically existing and non-existing forms, and finally, the no replies in the experimental conditions between the monolingually-raised and the heritage speakers’ groups.

**Table 4:** Distribution of alternative forms per group and per speakers.

	Raw number	HSs in the US	HSs Germany	Monolingual
Total alternative forms	281	241 (85,7%)	39 (13,8%)	1 (0,5%)
Morphologically existing/ grammatical forms	126	100 (40 speakers-79,4%)	25 (16 speakers-19,8%)	1 (1 speaker-0,8%)
Morphologically non-existing/ novel forms	125	115 (33 speakers-92%)	10 (5 speakers-8%)	–
No answer	30	26 (16 speakers-86,7%)	4 (3 speakers- 13,3%)	–

The second generalized linear mixed model (GLMM) was applied to analyze the use of novel forms among heritage speakers, excluding monolingually-raised participants from Greece. The model considered the effects of the country of residence, age of onset, and hours of education in Greek, with participants treated as a random effect. The model was fitted using a Poisson distribution following the same reasoning as the first model. The model included 122 observations, each representing a unique participant, with 117 residual degrees of freedom.

Regarding the random effects, the variance for the participant intercept was estimated at 1.397, with a standard deviation of 1.182. This suggests some variability in the baseline (group of heritage speakers in Germany) use of novel forms across participants, which the model accounts for.

The fixed effects revealed several significant factors. The intercept, which represents the baseline use of novel forms for the reference group, was estimated at −0.6789, with a significant p-value (p =.019). This indicates that, on average, the baseline use of novel forms is lower. The effect in the USA was significant (p =.019), with an estimate of 0.8287, suggesting that heritage speakers in the USA use more novel forms compared to those in the reference country. The age of onset showed a negative relationship with the use of novel forms, this effect was marginally significant though (β = −0.2712, SE = 0.1524, z = −1.78, p =.075). Finally, the hours of education in Greek had a significant negative effect, indicating that more education in Greek is associated with a lower use of novel forms (β = −0.467, SE = 0.2177, z = −2.150, p =.031).

Table 5 presents the estimated coefficients, standard errors, z-values, and p-values for the fixed effects, including country, age of onset, and hours of education in Greek, as well as the random effects for participants. Asterisks indicate significance levels and more precisely p < 0.05 values are given by one asterisk (*).

**Table 5:** Generalized Linear Mixed Model Results for the Use of Novel Forms Among Heritage Speakers.

FixedEffects	Estimate	Std.Error	z value	p-value
Intercept	−0.6789	0.2901	−2.341	0.019 *
Country	0.8287	0.3558	2.329	0.019 *
Age of onset to the majority language	−0.2712	0.1524	−1.780	0.075
Hours of education in Greek	−0.4679	0.2177	−2.150	0.031 *

In order to make the result section more transparent and the analysis of the verbal forms more comprehensive for the reader, we separate this section into two subsections. The first subsection analyses the results for the morphologically existing forms, while the second subsection for the morphologically non-existing forms. The objective of keeping separate the morphologically existing verbal forms that were produced by our heritage speakers is that these verbal forms should be treated differently from the non-existing ones. On the one hand, it is easy to detect which features seem to be re-organized in the morphologically existing verbal forms, while on the other hand, a different categorization scheme should be applied for the morphologically non-existing verbal forms.

### Results from morphologically existing forms

Beginning with the morphologically existing forms, as seen in Table 4 these are 125 instances found in heritage speakers’ data and one in a monolingually-raised speaker’s data. In S4 Appendix the distribution of the morphologically existing forms is also presented in more detail. These forms exhibit non-targeted features regarding aspect, tense, voice, mood, and phi-features. Table 6 presents the total score of the re-organized features detected in the morphologically existing verbal forms which the speakers produced. As seen in the examples (10–16) below, the verbal forms produced by the heritage speakers and one monolingually-raised speaker exhibit either one or a combination of untargeted verbal feature(s). Thus, one point was given for every untargeted feature and each verb could garner up to six points. Table 6 exhibits the re-organized features elicited from the task across heritage speaker groups. According to Fig 1, we would expect the voice feature to be more prone to change compared to aspect, but as long as our task doesn’t target any verbal forms in non-active voice, the next feature that is expected to be prone to change is the aspect. The aspectual feature is adhered to the root, and it can cause morpho-phonological changes in the case of [+PFV]. Tense appears also to be inconsistent as the augment which is needed to facilitate the stress in the verbs of 1^st^ CC is located outside of the right confines of the verbal form and additionally, tense morphemes appear after the aspectual morpheme and before the inflectional ending.

**Table 6:** Re-organized features in morphologically existing verbal forms across heritage speakers groups.

Features	Re-organized features in Heritage speakers in the US Raw number- Score (/126)	Re-organized features in Heritage speakers in Germany Raw number- Score (/126)
Number	36 (28,5%)	11 (8,7%)
Aspect	37 (29,3%)	10 (7,9%)
Tense	33 (26,2%)	9 (7,1%)
Person	21 (16,6%)	5 (4%)
Voice	7 (5,5%)	2 (1,6%)
Mood	3 (2,3%)	–

The first observation in Table 6 is that heritage speakers in the US re-organize more frequently the verbal features compared to heritage speakers in Germany. The two competing and most inconsistent features in heritage speakers’ productions are firstly the number, which is encoded in the inflectional suffix that denotes reflected-V agreement and secondly the aspect that usually causes morpho-phonological changes in the stem. It seems that aspect is more challenging for heritage speakers in the US while number for heritage speakers in Germany. In the hierarchy of features, the re-organization of tense comes in the third place and followed by the person, the voice and the mood in both groups.

Motivated by Table 6, we investigated further the aspectual feature by juxtaposing the expected aspect with the non-target aspect, as the primary focus of this study is the aspectual feature. All non-target aspectual features are found to be in 47 verbs. In 30 of them, the expected aspect was the IPFV, and in 17 of them the PFV. Considering that the PFV conditions were only 10 in the task while the IPVF were 20, we can observe a slight tendency of heritage speakers performing a bit more inaccurately in the PFV conditions. Regarding the tense feature, the competing tense was the present which was found in 36 verb forms, while 6 answers are formed in the perfect tense. None of the speakers produced any future tense. Moving on to voice, the competing counterpart that was detected in the 9 non-target verb forms was the non-active. Concerning the number, we observed a tendency to produce the verbs in the singular rather than in the expected plural. With respect to the person feature, the picture is mixed because from the 26 non-target answers 12 of them appeared in 1^st^ person, 7 of them in the 2^nd^ and 7 of them in the 3^rd^. Finally, instead of the targeted indicative mood, 2 verb forms in imperative and 1 in subjunctive are observed.

To better illustrate the non-target forms observed in our data we begin by presenting examples with the phi-features. Example (10) exhibits only the untargeted feature of number while example (11) exhibits the combination of the untargeted features of number and person.

**Table:** 

(10)	Expected form	màlon-e	GRmo17FG
		fight.IPFV.[+PST]/ 3SG	
	Produced form	màlon-an	
		fight.IPFV.[+PST]/ 3PL	
(11)	Expected form	èpsin-e	USbi10MG
		grill.IPFV.[+PST]/ 3SG	
	Produced form	psìnat-e	
		grill.IPFV.[+PST]/ 2PL	

As the responses that indicate only aspectual mismatches are categorized on their own the re-organization of the aspectual feature that appears in combination with other features as for instance, in example (12) with voice and tense is subject of the present study.

**Table:** 

(12)	Expected form	èha-s-e	Debi16FG
		lose.ACT.PFV.[+PST]/3SG	
	Produced form	hàne-s-e	
		be lost.NACT.IPFV.[−PST]/2SG	

A further case that accounts for aspect and tense re-organization is the production of perfect forms (example 13). These verbal forms are produced solely by one heritage speaker recruited in Germany. This speaker produced 6 cases of the Greek present perfect instead of the expected [+/- PFV] [+PST] verb forms. This indicates a possible interference from the majority German. As Klein [78] discusses about Modern German, the perfect (haben + participle) is identical to the [+PFV] interpretation, and there is no distinction between past and present perfect in German. The perfect is beyond the scope of the present study, and scholars argue for a separate treatment of it due to its distinct characteristics in different languages (for Greek, see [79]; for an overview, see [80]).

**Table:** 

(13)	Expected form	γìri-s-e	Debi70MG
		come back.PFV.[+PST]/ 3SG	
	Produced form	èhi γirìs-i	
		have.AUX come back.PFV.[−PST]/ 3SG	

Although all expected answers in all items should have been in the indicative mood, some participants produced other existing moods in Greek as below in example (14) with imperative.

**Table:** 

(14)	Expected form	èlin-an	USbi78FG
		solve.IPFV.[+PST]/3PL	
	Produced form	lìn-e	
		solve.IMP.2SG	

Moreover, several cases with tense inconsistencies can be found in our data. The most common strategy was to retain the present tense, although participants were explicitly told to assign to the given verb the past tense. As mentioned in the materials and methods section, participants practiced a few items before they began the main task, and in case of not assigning the past tense the elicitor highlighted this fact and participants were given a second chance. The surprising finding is that the verbs in which participants assigned the present tense were of both CC and not only of the 1^st^ CC where the augment is needed to facilitate the stress in the antepenultimate position. Even verbs of the 1^st^ CC that are trisyllabic and the application of the augment is redundant, some verbal forms are assigned in the present tense. Such a case is exhibited in example (15).

**Table:** 

(15)	Expected form	hòrev-e	USbi81MG
		dance.IPFV.[+PST]/3SG	
	Produced form	horèv-i	
		dance.IPFV.[-PST]/3SG	

Finally, in several cases, participants even produced and inflected different verbs from the targeted ones because of phonological similarity with the expected verbs (16). These cases are also included in this analysis scheme because these are morphologically existing forms despite the fact that the produced verb is ungrammatical in the given context or it is even infrequent in heritage productive vocabulary.

**Table:** 

(16)	Expected form	kolù-s-e	Debi02FG
		glue.IPFV.[+PST] /3PL	
	Produced form	kòla-s-e	
		entice.PVF.[+PST]/ 2SG	

In sum, phi-features, aspect and tense seem to be the most re-organized features while structuring a verbal form. A combination of several features is observed in most of heritage speakers’ productions of verbal forms in the elicitation task. In the following subsection, the focus is on the morphologically non-existing forms produced by the two groups of heritage speakers.

### Results from morphologically non-existing forms

Besides the morphologically existing verbal forms produced in the task, the heritage speakers produced non-existing and novel verbal structures, which are on the spotlight in the rest of this section. The analysis of these forms resembles the analysis of the existing forms by giving examples and justifying the relevant categorization. In order to observe some systematic patterns, we start by looking at the targeted aspect and how many non-existing forms have been produced in each targeted aspect per group (Table 7). The reader should keep in mind that the [+PFV] conditions in the elicited production task were only 10 while the IPFV conditions 20. What is observed is that the number of the non-existing forms, that is almost equal for every condition for the US group, while there is a slight tendency in the German group to produce more non-existing forms in the IPFV conditions.

**Table 7:** Distribution of non-existing forms per targeted condition across groups.

	Raw number	HSs in the US	HSs Germany
Non-existing morphologically ungrammatical forms	125	115	10
Target Perfective	40	38	2
Target Imperfective	85	77	8

Delving into the patterns that could be identified in these morphologically non-existing verbal forms, eight patterns that are presented in S5 Appendix Each novel verbal form produced by each speaker could get one point in several of the eight patterns. The combination of different patterns was common in plenty of the novel verbal forms, as we exhibit below in examples (17–28). The attempt to categorize the patterns in non-existing verbal forms is as unified as possible according to the eight different patterns and not per verbal features as it was done for the production of the morphologically existing but non-targeted verbs because it is hard to identify how heritage speakers mark each grammatical feature.

As it is presented in S5 Appendix, the most pervasive pattern in forming novel verbal morphological structures is the stem change for verbs of both CC. The other frequent patterns detected in our data are the CC change, the misplacement of stress, the erroneous placement or the omission of the augment, the redundant addition of the/s/ suffixation, and the non-past suffixation. In S2 Appendix an overview of all expected answers and the morphologically novel forms can be found.

The analysis of the alternative forms takes place in two ways. At first, we identify patterns common in several speakers’ productions and then we focus on patterns identified by single speakers in order to detect features restructuring in heritage grammars. Beginning with the first part of the analysis and the stem change classification detected in different speakers’ productions, examples (17) and (18) depict an erroneous stem change. Example (17) exhibits a verb form in which the stem ends in /v/. The speaker replaces the/v/ with a palatal/γ/ in the right border of the stem. According to the morphological rules, there seems to be no marker that indicates the perfective aspect, and thus, the form can be characterized as unmarked, identifying with the [−PFV] aspect. One could also think that the speaker changes the stem of the verb in order to mark the [+PFV] aspect but this remains ambiguous.

**Table:** 

(17)	Non-existing	δùle-		USbi69FG
	Existing target	δùlev-e		
		work.IPFV.[+PST]/ 3SG		

The pattern CC change indicates that the novel form produced resembles the verbs that are categorized in the other CC rather than in the CC that the verb prototypically belongs to. Similar to the verbs of 1^st^ CC, the novel form in example (18) exhibits an erroneous augment to facilitate the antepenultimate stress pattern. This form exhibits also a stem change pattern in addition to the erroneous augment placement and the change of CC. Regarding the aspectual marking it could be cautiously considered as unmarked if it is compared to verbs of the 1^st^ CC in IPFV aspect. Unlike verbs of the 2^nd^ CC, as the verb *for-ò* ‘wear’, which possess the exponent/s/ in both [-/+PFV] form, the [−PFV] past form of 1^st^ CC verbs do not get the exponent/s/ and additionally the stress is retained in the antepenultimate syllable.

**Table:** 

(18)	Non-existing	è-for-e	USbi70FG
	Existing target	fòra-g-e	
		wear.IPFV.[+PST]/ 3SG	

Moreover, example (19) indicates another novel verbal form combining a stem change pattern, a CC change and a stress change pattern. Although in verbs of 2^nd^ CC the exponent/s/ is added in the stem of both [-/+PFV] past tense forms, in this case the exponent is absent. The verb possibly carries the value of [+PFV] as the stress pattern resembles the PFV forms.

**Table:** 

(19)	Non-existing	pònet-e	USbi10MG
	Existing target	ponù-s-e	
		hurt.IPFV.[+PST]/ 3SG	

Another example of stem change, CC change, and omission of augment can be seen in (20). The verb *ràv-o* ‘sew’ belongs to the 1^st^ CC, but the addition of the morphemes/a/ and/γ/ between the root and the inflectional ending are characteristic of the [−PFV] past verbal forms of the 2^nd^ CC. Although the antepenultimate stress is retained, the augment is absent from this novel form.

**Table:** 

(20)	Non-existing	ràva-γ -e	USbi49MG
	Existing target	èrav-e	
		sew.IPFV.[+PST]/3SG	

One more pattern observed is the combination of the stem change and the stress placement is the/s/ suffixation. In example (21) we notice several similar novel verbal forms produced by different heritage speakers in the US. In all these forms, the vocalic element in the right boundary of the stem is either/i/ or/a/ and not the targeted/u/. Moreover, in the 3^rd^ novel form listed here, namely *pònakse,* we notice an extra addition to the stem which is the velar consonant/k/. The stress pattern in the antepenultimate syllable identifies with the stress pattern in the [+PFV] past forms of this CC.

**Table:** 

(21)	Non-existing	pòni-s-e/ pòna-s-e/ pònak-s-e/ pùna-s-e	USbi53FG/ USbi07FG/ USbi49MG/ USbi65FG
	Existing target	ponù-s-e	
		hurt.IPFV.[+PST]/ 3SG	

The next pattern noticed is the combination of stem change, the infelicitous addition of the/s/ suffixation and the CC change. These novel forms exhibit the construction of verbs categorized under the 2^nd^ CC, while the verb *ràvo* ‘sew’ belongs to the 1^st^ CC as mentioned before. Furthermore, considering that this verb is structured according to the 2^nd^ CC the addition of the exponent/s/ should be present in both [+/−PFV] forms. For the verbs of the 1^st^ CC though, the addition of the exponent/s/ is ungrammatical in [−PFV] forms. The stress pattern indicates that this novel form could denote the [+PFV] aspect in verbs of 2^nd^ CC as it is retained in the antepenultimate syllable, but it is unclear whether the speaker would place the stress in that syllable in order to assign the [−PFV] aspect. In short, the augment should facilitate the stress instead of changing the stem by adding one more syllable with the erroneous vocalic element/i/.

**Table:** 

(22)	Non-existing	ràvi-s-e/ ràvip-s-e	DEbi11FG/ DEbi73MG
	Existing target	èrav-e	
		sew.IPFV.[+PST]/3SG	

One clear pattern detected in our data is the misplacement of the stress (example 23). The speaker has correctly assigned the [−PFV] aspect indicated by the vocalic element/u/ and the/s/ suffixation, but she placed the stress in the antepenultimate syllable instead of the penultimate.

**Table:** 

(23)	Non-existing	pòn-u-s-e	USbi19FG
	Existing target	ponù-s-e	
		hurt.IPFV.[+PST]/3SG	

A further pattern concerning the omission of the augment, the stress misplacement and the CC change can be found in example (24). What we can detect in this novel verbal form is firstly the characteristic combination of a vowel, although not the targeted one, and the morpheme/γ/ which resembles the construction of [−PFV] verbal forms of the 2^nd^ CC. Secondly, two remarks can be made regarding the stress. The first remark is that the stress is placed before the antepenultimate syllable which indicates a reorganization of the phonological domain of the speaker and the second one is that the stress is not hosted by an augment.

**Table:** 

(24)	Non-existing	ràvoi-γ-e	USbi78FG
	Existing target	èrav-e	
		sew.IPFV.[+PST]/ 3SG	

Another not so frequent pattern observed in our data is the marking of NAct voice (example 26). Although the conditions of the task require only verbal forms in active voice as we mentioned in the description of the task design, a few participants assigned the NAct voice in the novel verbal forms similar to what they did for the existing verbal forms. The production of verbal forms bearing the NAct voice could be related to the argument of Oikonomou & Alexiadou [81], who support that speakers are given the opportunity to derive novel interpretations under the rule in example (25). Perhaps Greek heritage speakers productively use this rule by applying the NAct voice instead of canonical active. Thus, our assumption is that some speakers generalize the voice feature in favor of a morphological simplification. Regarding the aspectual feature in these novel forms we consider them as unmarked bearing the [−PFV] aspect.

**Table:** 

(25)	[VoiceP -external argument [XP_AGENT_ [vP DP}]]		
(26)	Non-existing	lin-ùmaste/ lìn-ante	USbi96MG/ USbi69FG
	Existing target	è-lin-an	
		solve.IPFV.[+PST]/ 3PL	

Finally, the least frequent pattern observed in our data, which is the dialectal form is exhibited in example (27). Although the forms listed under this pattern are not described as standard forms in the grammar of Modern Greek, these are spoken variants of Greek verbs found in different rural and/or isle areas.

**Table:** 

(27)	Non-existing	ì-fer-e	USbi25MG
	Existing target	è-fer-e	
		bring.PFV.[+PST]/ 3SG	

In order to investigate further the productions of the speakers’, we delve into individual speaker productions to identify common patterns in their responses (see S5 Appendix). Focusing on the non-past suffixation patterns, we observe inconsistencies regarding the tense marker. In example (28) the speaker places an erroneous augment which is an indicator of past tense marking. In addition to these patterns, the stress placement in the ultimate syllable is unexpected. Concerning the aspectual marking, we consider this as an unmarked novel form bearing the [−PFV] aspect. It should be noted here that the same Greek-American speaker produces most of his answers according to this pattern. An overgeneralization of the augment and the non-past suffixation are detected in almost all his answers, namely 28 out of 30, regardless of the targeted condition.

**Table:** 

(28)	Non-existing	eγel-à		USbi79MG
	Existing target	γèlaγ-e/ γ elù-s-e		
		laugh.IPFV.[+PST]/3SG		

Another Greek heritage speaker in the US with the code Usbi07FG produced 9 morphologically non-existing forms in which a stem change pattern is detected. More precisely, in example (29) in these 4 verbs of the 2^nd^ CC the vocalic element is changed.

**Table:** 

(29)	Non-existing	pùlase/	mìlase/	Usbi07FG
		pònase/	pònise	
	Existing target	pùlise/	mìlise/	
		ponùse/	ponùse	
		sell.PFV.[+PST]/3SG/ talk.PFV.[+PST]/3SG/ hurt.IPFV.[+PST]/3SG/ hurt.IPFV.[+PST]/3SG		

To support further the restructuring of verbal features in heritage grammars we provide example (30) from the speaker Usbi10MG whose productions point to a CC change. The 4 verbal forms below belong to the 1^st^ CC and the speaker adds the characteristic -aγ- between the root and the inflectional ending to denote the [-PFV]. The addition and adjustment of the vocalic element based on the aspectual value that it carries is also characteristic of verbs belonging to the 2^nd^ CC.

**Table:** 

(30)	Non-existing	lìnaγe/	δiòhnaγe/	Usbi10MG
		máluse/	malònise	
	Existing target	èlinan/	èδiokse/	
		málone/	málose	
		solve.IPFV.[+PST]/3PL./ send_away.PFV.[+PST]/3SG/ fight.IPFV.[+PST]/3SG/ fight.PFV.[+PST]/3SG		

Another pattern detected in some speakers is the tendency not to apply the augment to facilitate the trisyllabic stress rule as seen in example (31a-b). All verbs belong to the 1^st^ CC and require the addition of the augment regardless the [+/−PVF] aspect.

**Table:** 

(31a.)	Non-existing	lìhnikse/ rávise	Usbi56FG
	Existing target	è-linan/ èrave	
		AUG.solve.IPFV.[+PST]/3PL/ AUG.sew.IPFV.[+PST]/3SG	
b.	Non-existing	lionise/ psinùse	Usbi60FG
	Existing target	è-linan/ è-psine	
		AUG.solve.IPVF.[+PST]/3PL/ AUG.grill.IPFV..[+PST]/3SG	

Examples (28–31) show how the productions of individual speakers can point to certain patterns even the experimental items were only 30 with different types of verbs. Participants tend to restructure the morphological properties of the two CCs and the past tense morphology, either by using non-past suffixation or by being ambivalent about the presence or the absence of the augment/e/.

To identify whether the frequency of the verbs of the experimental items affected heritage speakers’ productions we searched how frequently these verbs appear in the online available material of the Corpus of Spoken Greek [82]. The frequency per ‰ tokens can be seen in S3 Appendix. Unfortunately, some of the verbs used in the elicited production task could not be found in the Corpus of Spoken Greek and thus, we performed a judgment task with monolingually-raised speakers. 37 adult participants born and raised in Greece (mean age 31;8) were asked how frequently they use or hear the verbs given. Their task was to rate the verbs in a scale from 1 (less frequent) to 10 (most frequent). The result is that monolingually-raised speakers rated all verbs with a score equal or above 5, meaning that all verbs in the experimental conditions are frequently used verbs in a monolingually-raised speaker’s repertoire.

In sum, heritage speakers produce some deviant forms in the elicited production task targeting [+/-PFV] aspect. These verbal forms are categorized either as existing or non-existing/ novel morphological forms based on the morphological features that they bear. The existing ones are analyzed in terms of voice, aspect, tense, and phi-features, while the non-existing verbal forms in terms of common patterns such as stem change, CC change, stress placement, erroneous augment,/s/ suffixation, non-past suffixation and dialectal variants. Greek heritage speakers in the US produced many more novel verbal forms indicating a divergent performance in comparison to heritage speakers in Germany. Having shown the different patterns observed in existing and non-existing/ novel verbal forms, we proceeded with the discussion, drawing some conclusions based on the RQs that were formulated.

## Discussion

This study provides an overview of novel morphological forms produced by different groups of Greek heritage speakers elicited through an oral task targeting the feature of aspect. Due to the complexity of Greek verbal morphology, the production of non-targeted forms was high, especially in the group of heritage speakers in the US. Although aspect is one of the first and well-investigated phenomenon in heritage language research, the focus of previous studies in Greek was its acquisition, the semantic distinction of the aspectual values and the telicity of the verbal phrase (VP), and the accuracy rates of the speakers’ performance (for acquisition see [22,24,25], for telicity see [83–84], for accuracy see [63,64,85]). The novelty of this study lies in the investigation- to the maximum possible extent- of non-targeted forms produced by the two heritage speaker groups in an elicited production task. Based on previous literature, three RQs with the respective hypotheses and predictions are derived. To answer these questions, the non-targeted verbal forms detected in the data are categorized as morphologically existing and morphologically non-existing verbal forms. The focus of the analysis is twofold. On the one hand, the analysis aims to identify which verbal features are prone to change in the morphologically existing verbal forms and, on the other hand, to explore systematic patterns identified in these non-existing novel forms. Below each RQ is addressed in turn.

According to RQ1 and H1, we predicted that heritage speakers will produce emerging and non-canonical patterns deviating from monolingually-raised speakers based on the Bottleneck Hypothesis proposed by Slabakova [47] and extended by Mikhaylova [49–50] to heritage speakers regarding the difficulties during the acquisition of functional morhology. Because Greek has concatenative verbal morphology heritage speakers face hinderances while acquiring morphological features rather while acquiring syntactic ones. The present study examines participants’ skills only on functional morphology via an elicited production task. The same participants have taken part in other experiments as well, such as in narration tasks. In the publication by Alexiadou et al. [86] the syntactic property of subject-verb agreement is explored concluding that verbal agreement in both groups of heritage speakers are almost error-free besides scarce evidence concerning person and number agreement in heritage speakers in the US in contrast to the error-prone nominal agreement. This is important to be mentioned under the scope of the Bottleneck Hypothesis proposed in RQ1 which compares the perforance of speakers in syntax and morphology. Furthermore, we pose the question of whether the heritage speaker groups differ from monolingually-raised speakers in terms of production of non-targeted patterns. To answer RQ1 is useful to look at Table 4. The two heritage speaker groups indeed produce alternative answers compared to monolingually-raised controls. These alternative verbal forms are categorized as morphologically existing and non-existing verbal forms. In general, both heritage speaker groups produced non-targeted answers in contrast to the monolingually-raised group. The generalized linear mixed model reveals that the monolingually-raised group differs significantly from the two heritage groups as reported in the results section. Heritage speakers in the US produce the most alternative verbal forms, as seen in Fig 2 and the high distribution, including morphologically existing and non-existing verbal forms, compared to Greek heritage speakers in Germany. This finding can be well explained by the Bottleneck Hypothesis, according to which heritage speakers face challenges with the functional verbal morphology and probably the differences between Greek and English or German mechanisms of constructing the verbal aspect. Especially the differences in mapping the interpretations of grammatical aspect in Greek puzzle them more. As mentioned in the Introduction, the imperfective aspect bears two different interpretations, namely the habitual and the continuous one, corresponding to one identical verbal form while the perfective aspect yielding a completed event is identified with a distinct morphological form. The habitual and the continuous interpretations can be disambiguated with the help of lexical aspect, namely with adverbial phrases. The grammatical aspect in the English language which is in contact with Greek, differentiates both semantically and morphologically from Greek. It encodes either continuity or progressivity which is marked with the distinguishable -ing suffix on the verb (Smith [17]). This falls under the notion of imperfective aspect which is quite different from the imperfective aspect in Greek. Unlike Greek, habituality in English can be expressed periphrastically with the expressions *used to* and *would*, and also with the suffix -ed, which furthermore expresses the perfectivity. Finally, German which is also in contact with Greek lacks grammatical aspect (Sioupi [45]). All these different mappings and interpretations of aspect in a bilingual mind might lead to a different performance on aspect compared to the monolingually-raised group.

Addressing RQ2, we are interested in exploring differences between the two heritage groups concerning the production of novel morphological forms, either existing or non-existing. Firstly, the model revealed that the two heritage speaker groups differ significantly from each other regarding the production of non-targeted patterns as reported in the results section. The group that produced more novel forms is the heritage speaker group in the US. Beginning with the morphologically existing forms we can state that φ-features (person and number) and the aspectual feature are more prone to change. These verbal features are found to be re-organized in the elicited production task targeting the expression of aspect in specific environments. As predicted in P2, we detect that speakers tend to assign the singular number instead of the plural and the [−PFV] aspect instead of the [+PFV] in the morphologically existing forms. Both the singular number and the [−PVF] aspect are unmarked in Greek, and thus, mostly heritage speakers in the US tend to simplify the verbal forms and employ the default forms regardless of the experimental condition requirements. At this point it is worth to clarify that the default form in many languages coincided with the unmarked form. In our case both singular and [−PFV] aspect are defaults under this notion, but according to L1 research the default is the form that is acquired first during childhood. In Greek the [+PFV] aspect is the one that is acquired first despite its morphological marking. This could be explained by the combination of firstly the feature reorganization as hypothesized by Scontras et al. [28,51] and the representational economy and secondly an effect of the majority language English that does not have a rich verbal morphology like Greek. The profound difference between heritage speakers in the US and in Germany is illustrated in Table 7.

Turning to the second aspect of RQ2, heritage speakers produced several morphologically non-existing verbal forms, as shown in Table 4. Heritage speakers rearranged the verbal features, resulting in the production of non-existing novel forms. The analysis of these forms revealed systematic patterns applied to the verbs, as seen in S5 Appendix. A supportive argument for the production of morphologically non-existing verb forms come from Bompolas et al. [67] who state that the type of stem allomorphy itself is the factor that inhibits or facilitates the transparency of the verbal form. Having a closer look in S2 Appendix, the novel verbal forms produced are found in all types of verbs. Novel verbal forms are found for strong verbs that require the exponent/s/ in combination with phonological changes and for verbs that require either weak or strong allomorphy. This means that we find both regularization and irregularization patterns. Arguing in favor of the representational economy proposed by Scontras et al. [28,51], in our case heritage speakers tend to use the most simplified verbal form in terms of morphophonemic rules applied to the stem in order to express all features that are encoded in each Greek verb. Focusing on aspect, as seen in Table 7, heritage speakers overcorrect and overgeneralize the unmarked form, namely the [−PFV] aspect, something that Seaman [60] observed in Greek American speakers. The fact of overcorrecting the unmarked form signals that heritage speakers are aware of the morphological marking that [+PFV] aspect requires. In the case of past tense marking, many speakers omit or misplace the augment, which signals the reorganization of tense marking, leaving aside the case of the speaker who used the non-past suffixation in almost all his answers. A surprising finding but in line with previous literature is the feature of voice. As Merchant [87] states, the null feature of voice in NAct [+PST] verbal forms makes it easier for heritage speakers to produce less complex non-target NAct verbal forms instead of the expected active forms. Novel forms with NAct morphology are productive in Greek, see [81], which is reflected also in the productions of heritage speakers.

Regarding the regularization patterns observed in our data, Bonfatti Sabbioni [88] mentions that systematic differences are observed between the two competing linguistic systems in contact in bilingual children. These differences can be seen primarily in the grammatical domain. The volatility in children’s performance lies in the specific properties of each language in contact, and the deviant patterns observed represent an attempt to regularize the language-specific rules. In this view, heritage speakers who haven’t established the lexical cluster of the different verbal forms, resemble bilingual children’s performance in their heritage language. In addition to the patterns detected among several speakers, we systematicaly analyzed the productions of individual speakers in order to look for tedencies. Examples (28–31) show stem change and CC change patterns, discrepancies with the augment and the past suffixation.

The exploration of language interference patterns, which is posed in RQ3 is partially confirmed. Firstly, structures pointing to language interference are detected in the heritage group in Germany, as reported in the results section and illustrated with example (13), while such patterns are not observed in the group of heritage speakers in the US. Secondly, one single pattern of language transfer from the majority language German is observed regarding the production of perfect verbal forms. As we mentioned in the previous section, German doesn’t differentiate between present and past perfect. Plausibly, the speaker who produced the Greek perfect (have + participle) has assumed the meaning of the German perfect, which has different semantics from the Greek one [79]. Considering that only one speaker produced all verbal forms in the Greek present perfect we cannot draw any strong claims concerning salient transfer effects from the majority language. Moreover, transfer effects regarding the verbal features from the majority language and the morphological system of it, are not possible to be detected due to the task design. A targeted online experiment on verbal morphology might reveal possible cross-linguistic effects.

In corroboration to RQ2 and the production of morphologically non-existing forms, comes RQ4, which explores the factors compiling heritage speakers’profiles. As shown in Table 5, different linguistic variables correlate with the production of novel forms in each heritage group. As we report, the hours of education correlate with the novel verbal forms produced by the heritage groups, which is in accordance with previous literature [12]. An almost marginal correlation between the age of onset to the majority language and the novel forms is also observed for the heritage groups. In line with previous literature, the background linguistic data play an important role for the performance in the heritage language. According to our prediction, the model indicates as a strong predictor the formal bilingual education, meaning that the education in the heritage language can significantly improve the performance on the phenomenon under the scope of this study. Concerning the age of onset that doesn’t reveal any significant correlation with the aspectual forms supports Tsimpli’s [29] hypothesis on the acquisition of late-acquired phenomena. Verbal aspect is considered a ‘core’ grammatical phenomenon in the Greek language, even though it is a late-acquired one and it is not affected by factors such as register [89].

In relation to the background linguistic data of the heritage speaker group in the US, a surprising finding exhibited in Table 4 can be explained. The observation is that 33 heritage speakers in the US group produce more novel and morphologically non-existing verbal forms (115 verbal forms) compared to the 5 heritage speakers in Germany who produced only 10 of them. Given the infrequent input in Greek, either this comes from human interaction (current input) or from written/ electronic means (literacy practices), heritage speakers in the US performed poorly in the experimental task by creating novel verbal forms to mark the different verbal features. From a generative approach, Perez-Cortes et al. [90] suggest under the notion of “attrition” that grammars do not disappear completely especially not without effort. Instead, the grammar becomes increasingly challenging to utilize for generating and understanding sentences. This happens because grammatical elements lack activation and continually face competition from other grammars in contact as time passes. Thus, as the researchers propose, heritage speakers might have differential access to competing representations, especially in the verbal domain overextending a “defective verbal inflection”. This could be indeed the case for the adult heritage speakers who participated in the study, but the notion of attrition cannot apply to adolescent heritage speakers who still visit the Greek Saturday courses and interact with teachers and their peers. The argument about the non-establishment of lexical clusters might be partially the case for our heritage speakers. According to Yang [91–92], who investigated the English past tense in irregular verbs, the expansion of children’s developing vocabulary helps them to strengthen and associate the morpho-phonological rules and patterns with the irregular verbal paradigms. At this point it is worth mentioning that in heritage speakers’ productions phonological discrepancies are observed as well besides the grammatical ones. Some of the non-existing verbal forms do not comply with the trisyllabic stress rule. Thus, we can cautiously attribute our heritage speakers’ performance on non-existing verbal forms to the infrequent and non-qualitative input that they received in the past while acquiring Greek. The limited access to qualitative input alongside the contact with the majority’s competing grammar resulted in novel patterns. However, this needs systematic exploration of their baseline’s input, something which is beyond the scope of the present study.

In sum, this study offers a detailed analysis of novel verbal morphological forms that different groups of heritage speakers produce. Significant differences were found between the groups, and more precisely, the most novel and morphologically non-existing forms were elicited in the US group. The major deviance from the grammatical forms was observed in the category of verbs that require both a root allomorphy and the exponent/s/ to mark [+PFV] aspect.

To the knowledge of the authors, this study is the first one, which offers an analysis of non-targeted forms in heritage speakers’ grammar focused on verbal morphology and precisely on verbal aspect. The study offers insights into morphological patterns that heritage speakers use to encode the verbal features, analyzing verbs of different classifications according to the verbal paradigms and of different CC. Thus far, studies in the field of heritage languages have tested heritage speakers’ performance on aspect using production and comprehension tasks regarding their accuracy in the given environments, while an error analysis has never taken place before. The present study compares and groups the morphological non-canonical forms based on the tendencies observed regarding aspectual marking, cautiously suggesting why heritage speakers produce these deviant forms. Moreover, the advantage of the study is that it presents and analyzes data from two distinct heritage speaker communities, one in the US and one in Germany.

## Limitations

This exploratory study presents a first approach of a systematic analysis of morphologically novel forms, although it is based on an unequal set of verbs of 1st and 2nd CC, which have different degrees of morpho-phonological rules applied to them. A comparative account between the verbs in which strong suppletion is applied compared to verbs with weak suppletion should be conducted in future studies allocating the same number of verbs of 1^st^ and 2^nd^ CC in each category with a more morphology-oriented task. An open issue that should be taken into consideration is the quality and quantity of the language background variables such as the input from their baseline. In addition to that, the task design does not take into account the test-retest reliability by administering the same test twice over a period of time to the same sample of participants. The bias of preoccupying our participants though in the case of retaking the same task would be looming over them.

## Conclusions

In this study we explore novel morphological patterns elicited in a production task in Greek heritage speakers in Germany and in the US. Given that Greek has fused morphology and each verb marks the features of aspect, tense, voice and number/person agreement, it is evident from a production task that heritage speakers face difficulties with the verbal inflectional morphology. The main patterns observed in our data are: i. the re-organization of φ-features and of aspectual marking [+/-PFV], ii. the reorganization of verbal structures by changing the stem and the inflectional paradigm of the conjugation class these verbs belong to, and by misplacing the stress. These findings are in line with the Bottleneck Hypothesis [45,47,48], supporting that heritage speakers are challenged by functional morphology and with the representational economy by Scontras et al. [28], proposing that heritage speakers simplify verbal forms by reducing complex morpho-phonological features.

## Supporting information

S1 Appendix Test items. (PDF)

S2 Appendix Novel morphologically non-existing forms. (PDF)

S3 Appendix Frequency of morphologically non-existing forms. (PDF)

S4 Appendix Distribution of morphologically existing forms. (PDF)

S5 Appendix Distribution of non-existing forms. can be found in figshare repository with the following DOIs: 10.6084/m9.figshare.26862247. The data seat: 10.6084/m9.figshare.26831812. The speakers’ data compiling their linguistic profile: 10.6084/m9.figshare.26831803. The R script: 10.6084/m9.figshare.26831815
(PDF)

## Abbreviations

ACT: active
Agr: agreement
AUG: augment
CC: conjugation class
Infl: inflection
IPFV: imperfective
HSs: heritage speakers
NACT: non-active
PFV: perfective
PL: plural
PRS: present
PST: past
SBJV: subjunctive
SG: singular
VP: verbal phrase
