# Supplementary material for: Novel forms for the expression of aspect in heritage Greek across majority languages
Source: PLoS One. 2025 May 15;20(5):e0319154. doi: 10.1371/journal.pone.0319154 (PMC12080926; doi:10.1371/journal.pone.0319154)
Supplement: S1 Appendix — (PDF) [file pone.0319154.s001.pdf]

S1 Appendix

In this section the pilot and the experimental items are listed in Greek with the equivalent translation below.

Προτάσεις εξοικείωσης:
Pilot sentences:

0. Η μητέρα της Νίκης, όταν ήταν νέα, …..…..… τα χαρτιά στις φίλες της. (ρίχνω)
0. When Niki's mum was young, she ……… the fortune in cards to her friends. (tell)

0. Ο Πέτρος είπε ότι δεν …..…..… τίποτα για τα σκάνδαλα. (γνωρίζω)
0. Petros said that he didn't ……… anything about the scandals. (know)

Προτάσεις της κύριας δοκιμασίας.
Items for the main study.
1. Παλιότερα οι φίλοι μου …..…..… σταυρόλεξα κάθε μέρα. (λύνω)

1. In the past years my friends ……… crosswords every day. (solve)

2. Προχθές ο κύριος …..…..… το παλιό του αυτοκίνητο σ' ένα φίλο του. (πουλάω)

2. The man ……… his old car to his friend two days ago. (sell)

3. Χθες το παιδί …..…..… στην τάξη συνέχεια. (γελάω)

3. Yesterday the child ………. In the classroom all the time. (laugh)

4. Την προηγούμενη βδομάδα ο Πέτρος …..…..… πέντε πελάτες απ' το μαγαζί του. (διώχνω)

4. Last week Petros ……… of his store five customers. (kick out)

5. Όταν ήταν νέος …..…..… στο σπίτι πολύ αργά. (γυρίζω)

5. When he was younger he ……… home late. (come back)

6. Χθες όλη μέρα …..…..… το κεφάλι μου. (πονάω)

6. Yesterday my head ……… all day long. (hurt)

7. Χθες ο Κώστας ξαφνικά …..…..… τον αδελφό του πολύ απότομα. (σπρώχνω)

7. Yesterday Kostas suddenly ……… his brother hard. (push)

8. Πέρυσι κάθε Κυριακή η Μαρία …..…..… στο πάρκο. (πηγαίνω)

8. Maria ……… to the park every Sunday last year. (walk)

9. Το Σάββατο όλο το πρωί η Καίτη …..…..… μια έκθεση. (γράφω)

9. During the whole morning on Saturday Keti ……… an essay. (write)

10. Πέρσι τα Χριστούγεννα ο νονός μας δεν μας …..…..… κανένα δώρο. (φέρνω)

10. Last Christmas our godfather didn't ……… us any present. (bring)

11. Την προηγούμενη βδομάδα η Έφη δεν μου …..…..… ούτε μια φορά. (μιλάω)

11. Efi hasn't ……… to me at all last week. (talk)

12. Πέρσι ο Κώστας με …..…..… τηλέφωνο σχεδόν κάθε μέρα. (παίρνω)

12. Kostas ……. me every day last year. (call)

13. Χθες στο πάρτι της Ελένης ο Νίκος …..…..… συνέχεια. (χορεύω)

13. Yesterday at Eleni's party Nikos ……… constantly. (dance)

14. Όλη την προηγούμενη βδομάδα η Ελένη …..…..… το σπίτι της. (βάφω)

14. All last week Eleni ……… her house. (paint)

15. Χθες το πρωί η Μαρία μου …..…..… ένα πολύ αστείο μήνυμα. (στέλνω)

15. Maria …….. me a funny message yesterday morning. (send)

16. Όταν ήμουν παιδί, ο πατέρας μου με …..…..… συχνά. (μαλώνω)

16. When I was a child, my father ……… me frequently. (scold)

17. Χθες όλο το βράδυ η Άννα …..…..… πολύ στην κοιλιά. (πονάω)

17. Anna's belly ……… yesterday night. (hurt)

18. Χθες ο Τάσος …..…..… στο σπίτι στις 8. (γυρίζω)

18. Tasos ……… home yesterday at 8 o' clock. (come back)

19. Χθες η δασκάλα ξαφνικά …..…..… τον Ανδρέα. (μαλώνω)

19. The teacher suddenly ……… Antreas yesterday. (scold)

20. Πέρσι η μητέρα μου …..…..… το αυτοκίνητό της κάθε Σάββατο. (πλένω)

20. Last year my mum ……… her car every Saturday. (wash)

21. Χθες η Μαρία …..…..… την ίδια μπλούζα όλη τη μέρα. (φοράω)

21. Maria ……… the same blouse the whole day yesterday. (wear)

22. Όλο το Σαββατοκύριακο ο Γιάννης .... τα πλακάκια. (κολλάω)

22. Gianis ……… floor tiles the whole weekend. (cement)

23. Η γιαγιά μου πέρυσι το χειμώνα συχνά …..…..… φαγητά σε φούρνο με ξύλα. (ψήνω)

23. My grandmother last winter often ……… food in the wood-fired oven. (grill)

24. Χθες ο Πέτρος …..…..… συνέχεια στο γραφείο του. (δουλεύω)

24. Petros ……… constantly in his office yesterday. (work)

25. Χθες η Νάντια δεν …..…..… ούτε μία ώρα. (δουλεύω)

25. Nantia didn't ……… at all yesterday. (work)

26. Όταν ήταν στην Αμερική ο θείος μας, μας …..…..… δώρα κάθε Χριστούγεννα. (στέλνω)

26. When my uncle was in the US, he ……… us presents every Christmas. (send)

27. Όταν ήταν μικρός …..…..… πολύ συχνά με τον αδελφό του. (μαλώνω)

27. When he was young he often ……… with his brother. (fight)

28. Χθες η μοδίστρα …..…..… το φόρεμα όλη τη μέρα. (ράβω)

28. The dressmaker ……… the dress all day long yesterday. (sew)

29. Παλιά ο ψαράς …..…..… έξω από το σπίτι μας δυο φορές τη βδομάδα. (περνάω)

29. (In the past) the fisherman ……… to our house twice a week. (walk)

30. Χθες ο Άρης …..…..… το πορτοφόλι του στο λεωφορείο. (χάνω)

30. Aris ……… his wallet in the bus yesterday. (loose)
