# Supplementary material for: Novel forms for the expression of aspect in heritage Greek across majority languages
Source: PLoS One. 2025 May 15;20(5):e0319154. doi: 10.1371/journal.pone.0319154 (PMC12080926; doi:10.1371/journal.pone.0319154)
Supplement: S2 Appendix — (PDF) [file pone.0319154.s002.pdf]

S2 Appendix

Novel morphologically non-existing forms produced by HSs in the US and Germany

Expected verb		Meaning	Novel form
IPFV	PFV		
γìrize	γìrise	come back	γirnòtane<br>γirùse (appears twice)<br>eγirìzo (appears twice)
γèlage		laugh	èlaγe<br>eγelà<br>èγele
δùleve	δùlepse	work	δùleγe (appears twice)<br>eδulèvi<br>eδulèvo
èvafe		paint	evàfo
èγrafe		write	eγràfo
	èδiokse	send away/repel	δiòhnise (appears twice)<br>δiòhnaγe<br>èδiohe<br>eδiòhno
èlinan		solve	lìnante<br>elìno<br>lìnaγe<br>linòteran<br>lìhnikse<br>linùmaste<br>liònise
èperne		take	epèrno
èplene		wash	èplise<br>eplèni<br>eplèno
èrave		sew	ràvise (appears three times)<br>ràvaγe<br>ràvipse (appears twice)

			eràvo<br>ravòiγe
	èsprokse	push	spròhnage<br>èsprohno
èstelne	èstile	send	èstilne (appears eight times)<br>èstele (appears five times)<br>estèlno<br>èstelno<br>ìstile
	èfere	bring	èferni<br>efèrno<br>ìfere<br>èrfine
	èhase	lost	èhaze<br>ehàno
èpsine		grill	psinùse<br>psìnage
kolùse		glue	kòline<br>ekolào
màlone	màlose	fight/ scolded sb	emalòni<br>emalòno (appears twice)<br>malùse (appears four times)<br>màluse (appears five times)<br>malòthike (appears two times)<br>malònese (appears three times)<br>malònise<br>malonòmun<br>malònose<br>malòtane
	mìlise	talk	mìlase (appears twice)

			emilào
pèrnage		pass by	pèrnase (appears six times)<br>epernào
pìgene		go	epìγane<br>epiγèni
ponùse		hurt	pònete<br>pònise<br>pònakse<br>pùnase<br>eponà<br>pònuse<br>eponào
	pùlise	sell	pùlakse<br>pùlase<br>epùlise
fòrage		wear	fòrase (appears twice)<br>èfore<br>fùrage<br>eforà
hòreve		dance	ehorèvo
