# Supplementary material for: Novel forms for the expression of aspect in heritage Greek across majority languages
Source: PLoS One. 2025 May 15;20(5):e0319154. doi: 10.1371/journal.pone.0319154 (PMC12080926; doi:10.1371/journal.pone.0319154)
Supplement: S3 Appendix — (PDF) [file pone.0319154.s003.pdf]

S3 Appendix

Table I presents the verbs in which heritage speakers replied with an unexpected answer, and the aim of Table I is to exhibit in which verbs heritage speakers face difficulties in producing the targeted verbal form. The raw number and the frequency of the verbs for which morphologically existing and non-existing verbal forms were produced by heritage speakers in the US and Germany per condition are listed below. The raw number and consequently the frequency include also the ‘no reply’ that HSs gave as an answer. As seen in Table I the total number of the morphologically alternative forms is 281 answers including the existing, the non-existing and the ‘no answer’ replies. These are here are analyzed per targeted verbal form and condition. Furthermore, to capture the frequency of the targeted verbs, a corpus search was conducted in the online version of the corpus of Spoken Greek compilated with data from monolingual speakers (671.543 tokens) and in addition to this search a questionnaire is distributed to different monolingually-raised participants to assess the frequency of the targeted verbs in Greek. The latter research is conducted among 37 monolingually-raised participants (mean age 31;8, age range 19-39) in the form of a likert scale (1 less frequent- 10 most frequent). In Table II the metadata of the monolingually-raised participants who took part in the judgments are exhibited.

Table I: Distribution of alternative forms across verbs and the respective frequency alongside the frequency of these verbs in the corpus of Spoken Greek and the ratings of monolingually-raised speakers in an offline judgment task

Condition	Expected Verb	Raw number of morphologically alternative forms	Frequency per % in the elicited production task	Frequency of the lexical verb in the corpus of Spoken Greek per ‰	Ratings of monolingual speakers in an offline judgment task
PVF	pùlise ‘sold’	8	> 5 (6,34)	-	> 5 (7,51)
	gìrise ‘came back’	3	< 5 (2,38)	2,38	> 5 (8,78)
	dùlepse ‘worked’	3	< 5 (2,38)	1,04	> 5 (8,7)
	èdiokse ‘sent away/ repelled’	14	>10 (11,11)	-	> 5 (6,72)
	èsprokse ‘pushed’	7	> 5 (5,55)	-	> 5 (5,94)
	èstile ‘sent’	9	> 5 (7,14)	0,59	> 5 (9,54)
	èfere ‘brought’	7	> 5 (5,55)	0,59	> 5 (8,54)
	èhase ‘lost’	6	<5 (4,76)	1,48	> 5 (8,56)
	màlose ‘fought/ scold sb’	9	> 5 (7,14)	0,44	> 5 (7,72)
	mìlise ‘talked’	4	<5 (3,17)	13,4	> 5 (8,91)

IPFV continuous	gèlage 'was laughing'	4	<5 (3,17)	0.59	> 5 (7,4)
	dùleve 'was working'	4	<5 (3,17)	as above in dùlepse 'worked'	> 5 (9,24)
	èvafe 'was painting'	4	<5 (3,17)	-	> 5 (6,72)
	ègrafe 'was writing'	2	<5 (1,58)	1,63	> 5 (8,45)
	èrave 'was sewing'	17	>10 (13,49)	-	= 5 (5)
	kolùse 'was gluing'	13	>10 (10,31)	1,04	> 5 (6,45)
	ponùse (appears twice in the items) 'was hurting'	14	> 5 (5,55)	0,14	> 5 (7,62)
	fòrage 'was wearing'	7	> 5 (5,55)	1,48	> 5 (6,08)
	hòreve 'was dancing'	3	<5 (2,38)	0,14	> 5 (6,86)
IPVF habitual	èlinan 'were solving'	56	>10 (44,44)	-	> 5 (6,16)
	èperne with the meaning 'was calling'	5	<5 (3,96)	16,08	> 5 (7,86)
	èplene 'was washing'	6	<5 (4,76)	-	> 5 (7,81)
	èstelne 'was sending'	12	> 5 (9,52)	as above in èstile 'sent'	> 5 (7,81)
	èpsine 'was grilling'	8	> 5 (6,34)	-	> 5 (6,21)
	màlone (appears twice in the items) 'was scolding sb'	27	>10 (10,71)	as above in màlose 'fought/ scold sb'	> 5 (6,72)
	pèrnage 'was passing by'	14	>10 (11,11)	1,78	> 5 (5,7)
	pìgene 'was going'	4	<5 (3,17)	6,4	> 5 (8,13)

	gìrize ‘was coming back’	10	> 5 (7,93)	as above in gìrise ‘came back’	> 5 (6,91)
Total number of alternative forms		281			

Table II. Meta-linguistic data of monolingual speakers participated in the judgment task.

		Participants
Highest education degree	High school/ technical school	4
	Technical education	4
	University degree	18
	Master degree	11
Region of Residence	Attica	11
	Thessaloniki	21
	Other regions of Northern Greece	5
