# Supplementary material for: Novel forms for the expression of aspect in heritage Greek across majority languages
Source: PLoS One. 2025 May 15;20(5):e0319154. doi: 10.1371/journal.pone.0319154 (PMC12080926; doi:10.1371/journal.pone.0319154)
Supplement: S4 Appendix — (PDF) [file pone.0319154.s004.pdf]

S4 Appendix

Distribution of morphologically existing forms per participant

Anonymized Participant	Categorization of alternative forms according to the type of erroneous feature/s produced by HSs in Germany (existing verbal forms)					
	Wrong verb	Number	Person	Aspect & Person	Aspect, Voice & Person	Tense
Debi02FG	1					
Debi06FG		1				
Debi07FG		1				
Debi08MG		1				
Debi11FG		2				
Debi12FG		1				
Debi16FG	1			1	1	
Debi17FG			1			
Debi19MG			1			
Debi20FG		1				
Debi21MG		1				
Debi22FG		1	1			
Debi57MG		1				
Debi64FG						1
Debi70MG						6
Debi72MG		1				

Morphologically existing forms produced by HSs in the US

Anonymized Participant	Categorization of alternative forms according to the type of erroneous feature/s produced by HSs in the US (existing verbal forms)														
	Wrong Verb	Number	Person	Aspect & Number	Aspect & Person	Number & Person	Tense	Tense & Aspect	Mood	Aspect, Voice & Person	Tense & Person	Tense & Number	Tense, Number & Person	Voice	Voice & Person
Usbi01FG		1													
Usbi02FG	1														
Usbi07FG	2	1													
Usbi08FG							1								
Usbi09MG				1											
Usbi10MG	2	2		1		1			1	1		1			
Usbi14MG	1														
Usbi17MG		1												2	

Usbi20FG		1													
Usbi24FG							1								
Usbi28MG	1														
Usbi29MG				1											
Usbi31FG				1											
Usbi32FG		1													
Usbi49MG		1													
Usbi50FG	3	1			2										
Usbi51MG	1														
Usbi52FG	1	1													
Usbi53FG	1														
Usbi55MG	1														
Usbi56FG	1														
Usbi64FG		1													
Usbi65FG	1														
Usbi66MG		1	1												
Usbi67MG		1													
Usbi68MG	1	1													
Usbi70FG		1			1										
Usbi73FG		1													
USbi74MG											1				
Usbi78FG	1								1						
USbi79MG	1														
USbi81MG	2						16	8	1				1		
Usbi85MG		2													
USbi86FG		1													
Usbi87MG	2		1		1										

Usbi8 8FG	1														
Usbi8 9FG		1		1											
Usbi9 2FG				1										1	
Usbi9 6MG	1		1	1											
Usbi9 7FG															1
