# Supplementary material for: Novel forms for the expression of aspect in heritage Greek across majority languages
Source: PLoS One. 2025 May 15;20(5):e0319154. doi: 10.1371/journal.pone.0319154 (PMC12080926; doi:10.1371/journal.pone.0319154)
Supplement: S5 Appendix — can be found in figshare repository with the following DOIs: 10.6084/m9.figshare.26862247. The data seat: 10.6084/m9.figshare.26831812. The speakers’ data compiling their linguistic profile: 10.6084/m9.figshare.26831803. The R script: 10.6084/m9.figshare.26831815 (PDF) [file pone.0319154.s005.pdf]

S5 Appendix

Distribution of morphologically non-existing forms per HS in Germany

Anonymized Participant	N of non-existing forms produced
Debi11FG	1
Debi13MG	1
Debi16FG	4
Debi65MG	1
Debi73MG	3
Total	10

Patterns detected in the morphologically non-existing verbal forms

Anonymized Participant	Non-existing verb form produced	Categorization of alternative forms according to different patterns produced by HSs in Germany (non-existing verbal forms)				
		Stress Misplacement	/S/ suffixation	CC change	Stem change	Augment
Debi11FG	rávise		×	×	×	×
Debi13MG	malùse	×	×	×	×	
Debi16FG	δiòhnise			×	×	×
Debi16FG	èstele				×	
Debi16FG	psinùse	×	×	×	×	×
Debi16FG	èstele				×	
Debi65MG	pèrnase		×		×	
Debi73MG	èplise		×		×	
Debi73MG	psìnaɣe			×	×	×
Debi73MG	rávipse		×	×	×	×

Distribution of morphologically non-existing forms per HS in the US

Anonymized Participant	N of non-existing forms produced
Usbi02FG	1
Usbi05MG	1
Usbi07FG	9
Usbi10MG	7
Usbi17MG	1
Usbi20FG	2
Usbi24FG	2
Usbi25MG	1
Usbi31FG	1
Usbi32FG	1
Usbi35FG	2
Usbi49MG	2
Usbi50FG	4
Usbi53FG	3
Usbi55MG	1
Usbi56FG	3

Usbi60FG	2
Usbi61MG	4
Usbi65FG	6
Usbi66MG	2
Usbi69FG	4
Usbi74MG	3
Usbi75MG	2
Usbi78FG	5
Usbi79MG	28
Usbi82FG	1
Usbi84FG	1
Usbi85MG	1
Usbi87MG	6
Usbi89FG	1
Usbi91FG	2
Usbi96MG	5
USbi97FG	1
Total	115

Patterns detected in the morphologically non-existing verbal forms

Anonymized Participant	Non-existing verb form produced	Categorization of alternative forms according to different patterns produced by HSs in Germany (non-existing verbal forms)							
		Stress Misplacement	/S/ suffixation	CC change	Stem change	Augment	Dialectal	Voice	Non past suffixation
Usbi02FG	malùse	×	×	×	×				
Usbi05MG	malòtane		×		×				
Usbi07FG	pùlase				×				
Usbi07FG	δiòhnise				×	×			
Usbi07FG	pònise	×			×				
Usbi07FG	èferni								×
Usbi07FG	mìlase				×				
Usbi07FG	èstele				×				
Usbi07FG	pònase	×			×				
Usbi07FG	èpleni								×
Usbi07FG	èfore			×	×	×			
Usbi10MG	lìnaγe			×	×	×			

Usbi10MG	pùlase				×				
Usbi10MG	δiòhnaγe			×	×	×			
Usbi10MG	máluse		×	×	×				
Usbi10MG	pònete	×	×		×				
Usbi10MG	malònise			×	×				
Usbi10MG	fùraγe				×				
Usbi17MG	γirnòtane							×	
Usbi20FG	èγele			×	×	×			
Usbi20FG	èstele				×				
Usbi24FG	malòthike							×	
Usbi24FG	malòthike							×	
Usbi25MG	ìfere					×	×		
Usbi31FG	γirùse		×		×				
Usbi32FG	èstilne				×				
Usbi35FG	γirùse		×		×				
Usbi35FG	spròhnaγe			×	×	×			
Usbi49MG	pònakse	×			×				
Usbi49MG	rávaγe			×	×	×			
Usbi50FG	pùlase				×				
Usbi50FG	epìgane						×		
Usbi50FG	fòrase		×						
Usbi50FG	ìstile		×		×				
Usbi53FG	pònise	×			×				
Usbi53FG	èstilne				×				
Usbi53FG	èstilne				×				
Usbi55MG	kòline	×	×	×	×				
Usbi56FG	lìhnikse		×	×	×	×			

Usbi56FG	rávise		×	×	×	×			
Usbi56FG	èhaze		×		×				
Usbi60FG	liònise		×	×	×	×			
Usbi60FG	psinùse		×	×	×	×			
Usbi61MG	máluse		×	×	×				
Usbi61MG	pònise	×			×				
Usbi61MG	rávipse		×	×	×	×			
Usbi61MG	pèrnase		×		×				
Usbi65FG	linòteran					×		×	
Usbi65FG	máluse		×	×	×				
Usbi65FG	pùnase	×			×				
Usbi65FG	máluse		×	×	×				
Usbi65FG	fòrase		×						
Usbi65FG	máluse		×	×	×				
Usbi66MG	èlaγe				×				
Usbi66MG	pònise	×			×				
Usbi69FG	lìnante					×		×	×
Usbi69FG	pòlise				×				
Usbi69FG	δùleγe				×				
Usbi69FG	δùleγe		×		×				
Usbi74MG	mìlase				×				
Usbi74MG	malùse	×		×	×				
Usbi74MG	èstilne				×				
Usbi75MG	kòline			×	×				
Usbi75MG	malùse	×		×	×				
Usbi78FG	pùlakse				×				
Usbi78FG	èδiohe		×		×				

Usbi78FG	èstele				×				
Usbi78FG	malònese							×	
Usbi78FG	rávoiγe	×		×	×				
Usbi79MG	epùlise						×		
Usbi79MG	eγelá	×							×
Usbi79MG	eδiòhno	×							×
Usbi79MG	eγirizo	×				×			×
Usbi79MG	eponá	×				×			×
Usbi79MG	èsprohno								×
Usbi79MG	epiγèni	×				×			×
Usbi79MG	eγráfo	×							×
Usbi79MG	efèrno	×							×
Usbi79MG	emiláo	×				×			×
Usbi79MG	epèrno	×							×
Usbi79MG	ehorèvo	×				×			×
Usbi79MG	eváfo	×							×
Usbi79MG	estèlno	×							×
Usbi79MG	emalòni	×				×			×
Usbi79MG	eponáo	×				×			×
Usbi79MG	eγirìzo	×				×			×
Usbi79MG	emalòno	×				×			×
Usbi79MG	eplèno	×							×
Usbi79MG	eforá	×				×			×
Usbi79MG	ekoláo	×				×			×
Usbi79MG	eδulèvi	×				×			×
Usbi79MG	eδulèvo	×				×			×
Usbi79MG	èstelno								×

Usbi79MG	emalòno	×				×			×
Usbi79MG	erávo	×							×
Usbi79MG	epernáo	×				×			×
Usbi79MG	ehάno	×							×
Usbi82FG	pèrnase		×		×				
Usbi84FG	èstilne				×				
Usbi85MG	èstilne				×				
Usbi87MG	èlino								×
Usbi87MG	èrfine				×				
Usbi87MG	malònese							×	×
Usbi87MG	malònose							×	×
Usbi87MG	malònese							×	×
Usbi87MG	pèrnase		×		×				
Usbi89FG	pèrnase		×		×				
Usbi91FG	pùlase				×				
Usbi91FG	pònuse	×							
Usbi96MG	linùmaste					×		×	×
Usbi96MG	èstilne				×				
Usbi96MG	èstilne				×				
Usbi96MG	rávise		×	×	×	×			
Usbi96MG	pèrnase				×				
Usbi97FG	malonòmun	×						×	
